# Structural and optical variation of pseudoisocyanine aggregates nucleated on DNA substrates

**DOI:** 10.1088/2050-6120/acb2b4

**Published:** 2023-01-31

**Authors:** Matthew Chiriboga, Christopher M Green, Divita Mathur, David A Hastman, Joseph S Melinger, Remi Veneziano, Igor L Medintz, Sebastián A Díaz

**Affiliations:** 1 Center for Bio/Molecular Science and Engineering Code 6900, U. S. Naval Research Laboratory, 4555 Overlook Ave. S.W. Washington, DC 20375, United States of America; 2 Department of Bioengineering. College of Engineering and Computing, George Mason University, 4400 University Drive, Fairfax, VA 22030, United States of America; 3 Department of Chemistry, Case Western Reserve University, 10900 Euclid Avenue, Cleveland, OH 44106, United States of America; 4 Electronics Sciences and Technology Division, U.S. Naval Research Laboratory, 4555 Overlook Ave. S.W. Washington, DC 20375, United States of America

**Keywords:** DNA nanostructures, dye aggregates, UV/Vis spectroscopy, fluorescence spectroscopy, circular dichroism, atomic force microscopy, energy transfer

## Abstract

Coherently coupled pseudoisocyanine (PIC) dye aggregates have demonstrated the ability to delocalize electronic excitations and ultimately migrate excitons with much higher efficiency than similar designs where excitations are isolated to individual chromophores. Here, we report initial evidence of a new type of PIC aggregate, formed through heterogeneous nucleation on DNA oligonucleotides, displaying photophysical properties that differ significantly from previously reported aggregates. This new aggregate, which we call the super aggregate (SA) due to the need for elevated dye excess to form it, is clearly differentiated from previously reported aggregates by spectroscopic and biophysical characterization. In emission spectra, the SA exhibits peak narrowing and, in some cases, significant quantum yield variation, indicative of stronger coupling in cyanine dyes. The SA was further characterized with circular dichroism and atomic force microscopy observing unique features depending on the DNA substrate. Then by integrating an AlexaFluor^™^ 647 (AF) dye as an energy transfer acceptor into the system, we observed mixed energy transfer characteristics using the different DNA. For example, SA formed with a rigid DNA double crossover tile (DX-tile) substrate resulted in AF emission sensitization. While SA formed with more flexible non-DX-tile DNA (i.e. duplex and single strand DNA) resulted in AF emission quenching. These combined characterizations strongly imply that DNA-based PIC aggregate properties can be controlled through simple modifications to the DNA substrate’s sequence and geometry. Ultimately, we aim to inform rational design principles for future device prototyping. For example, one key conclusion of the study is that the high absorbance cross-section and efficient energy transfer observed with rigid substrates made for better photonic antennae, compared to flexible DNA substrates.

## Introduction

The ability to maximize the range of exciton transport while minimizing energy loss has significant implications for the design of future nanoscale light harvesting, optoelectronic, and sensing applications [1–4]. One method of achieving this would be to densely pack dyes into strongly coupled aggregates such that excitations can be coherently delocalized through the partial or full length of the aggregate [5]. Coherently coupled aggregates enable exciton migration over discreet spatial distances with near unitary quantum efficiency [6, 7]. As a result, controlled dye aggregation has long been studied by chemists as a method of tuning the photonic and physical properties of the dyes and pigments in light harvesting devices [5, 8, 9]. An example of this coherent coupling phenomenon can be observed in the cyanine dye family and specifically the prototypical example, pseudoisocyanine (PIC) dye [8, 10]. In the mid-1930s, Scheibe *et al* [11] and Jelley [12] both independently observed interesting aggregation behavior from aqueous mixtures of PIC dye. Franck and Teller then further demonstrated that the aggregates required solvation and that their interesting photophysical properties were lost upon drying [13]. These dye aggregates, which became known as J-aggregates (J signifying Jelley), are characterized by bathochromic absorption shifts, increased quantum yields, and narrowed Stokes shifts, relative to the monomer [8]. The current consensus is that the observed changes in PIC dye subsequent to J-aggregation are the result of coherent coupling between the monomers [8, 11, 14, 15].

Prior to this discovery, the theory surrounding exciton behavior and transport was predominantly described from the perspective of isolated excitations generated in individual chromophores and transferred between dyes through weak electromagnetic (EM) coupling mechanisms. For example, this perspective is reflected in the contributions of Förster [16] and Dexter [17] who outlined energy transfer frameworks based on incoherent rate constants derived from Coulombic dipole-dipole coupling (through-space) and electron exchange (through-bond) mechanisms [18]. When considering J-aggregates on the other hand, closely packed monomers can generate collective excitations which respond in phase to an external EM field [7]. The dense packing and rigid alignment allow the monomer transition dipole moments (TDMs) to superimpose with the excited states then considered to be delocalized across multiple PIC molecules [14, 19]. This leads to optimal conditions for highly efficient energy transfer and other sought-after non-linear optical properties, such as cooperative radiative decay (superradiance) [14, 20, 21].

However, strongly coupled aggregates can also exhibit some variation and may display substantially different properties depending on monomer packing and orientation. For example, J-type aggregation occurs when the monomer TDMs are aligned head-to-tail [8, 15, 22, 23]. Alternatively, H-type aggregates, which exhibit reversed properties (hypsochromic absorption, emission quenching, *etc*), occur when the monomers are packed face-to-face and the TDMs stack in a parallel orientation [8, 22–24]. Notably, even with similarly packed monomers, Eisfeld and colleagues demonstrated that the size and shape of, in this case 2D aggregates, modified the superradiance [25]. Importantly, the monomer packing is highly sensitive and closely related to the environmental conditions imposed on the dye system [8]. This has led to the development of various strategies for controlling dye aggregation ultimately aimed at directing the monomer packing-type and the subsequent optical properties [5]. Simplistic strategies have been proposed, such as fine-tuning buffer components such that conditions are optimal for driving spontaneous aggregate self-assembly [26–29]. Also, more intensive methods have been investigated, such as synthetic dye modification or tethering dyes to cofactors which may favor aggregation or restrict the dye’s ability to adopt certain orientations [30–36].

Though early investigations of the interactions between generic cyanine dyes and DNA templates were pioneered by the Armitage group [37–41], more recently the Bathe group described a controlled aggregation strategy specifically for PIC dye using a sequence specific DNA scaffold [30, 31]. These studies demonstrated that duplex DNA can spontaneously template PIC aggregates under certain conditions. Principally, DNA-bound aggregate formation requires a DNA template with a non-alternating poly(AT) sequence (AT-track) [30–33]. Remarkably, these DNA-bound aggregates, though distinct from solution-based J-aggregates, have been observed to retain J-aggregate-like properties such as superradiance [30, 31]. These observations, reminiscent of J-aggregates, have led to these DNA-based PIC aggregates being colloquially referred to in the field as J-bits [30, 31, 33]. Available evidence suggests that within J-bits, a fraction of the monomers preserve some degree of coherent coupling. Specifically, computational modeling supported by some spectroscopic measurements indicate J-bits can form in AT-tracks as short as 8 base pairs (bp) with coherence lengths (*N*_coh_) reaching upwards of 3 PIC molecules [22, 30, 31]. Furthermore, when donor and acceptor fluorophores were tethered at opposite ends of a DNA scaffold, J-bits were able to act as an exciton relay through Förster resonance energy transfer (FRET) [30–33]. In summary, a J-bit is a short PIC (3–8 nm in length) aggregate specifically localized in AT-track DNA with spectral characteristics similar to J-aggregates. These results have been viewed as initial steps towards a bottom-up strategy for the rational design of strongly coupled excitonic circuits [30]. However, this approach currently relies on the inherent propensity of PIC dye to preferentially form a certain type of aggregate at high excess concentration ratios relative to the DNA template, which lacks explicit control over the dye packing/molecular orientation and, by extension, the observed optical properties. The ability to directly control aggregation would be more feasibly translatable to a larger space of potential applications, such as fluorescence sensors, optical switches, and input/output interfaces [1–4, 42, 43].

Here we show that DNA-nucleated PIC aggregates have properties which correlate to different molecular structures and are directed by changing the DNA scaffold. To achieve this, we formed PIC aggregates through heterogeneous nucleation by mixing dissolved PIC dye with various DNA nanostructures ranging from a rigid DX-tile [44] to more flexible DNA duplex (dsDNA) or single strand DNA oligonucleotide (ssDNA). Although the aggregates we formed required elevated excess of PIC dye relative to previously reported J-bits, they exhibited sharper and brighter fluorescence peaks as well as longer *N*_coh_ (as discussed in the Results section). Therefore, we refer to aggregates formed by this approach as super aggregate (SA), though we note SA formed with different DNA substrates result in unique properties. Importantly, controls using deoxynucleotide triphosphate (dNTP) salts or lacking nucleic acids all together, did not result in SA formation, signifying that DNA oligos are crucial to formation of the aggregates. Although, J-bits require a lower bulk dye concentration, the fully formed SAs exhibited excitations more in line with solution-based J-aggregates when mixed with identical DNA substrate [8, 23]. When in proximity to an AF energy transfer acceptor, SA nucleated DX-tile DNA resulted in increased AF emission, with the level of increase depending on the DNA sequence. Alternatively, flexible ss- and dsDNA substrates resulted in emission quenching. Complementary circular dichroism (CD) and atomic force microscopy (AFM) characterizations both indicated distinctions in the way each substrate and subsequent dye aggregate incorporates the individual PIC molecules. Cumulatively, these results suggest modification of the DNA substrate results in significant changes to how the DNA and companion dye molecules are integrated into larger form PIC aggregates.

## Materials and methods

### Materials

ssDNA oligonucleotides and AlexaFluor^™^ 647-labeled ssDNA oligonucleotides were purchased from Integrated DNA Technologies Inc. Full oligonucleotide sequences can be found in the accompanying Supporting Information (SI) file. Ultrapure equimolar deoxynucleotide triphosphate (dNTP) sodium salt solution was purchased from New England Biolabs Inc. (Catalog #N0447S) as an equimolar mixture of 10 mM dATP, dCTP, dGTP and dTTP giving a final dNTP concentration of 40 mM. AlexaFluor^™^ 647 C_2_ maleimide (Free AF) was purchased from ThermoFisher Scientific (Catalog #A20347). All other chemicals including 97% 1, 1′-diethyl-2, 2′-cyanine iodide (PIC, CAS #:977-96-8); molecular biology-grade water; sodium chloride (NaCl); magnesium chloride (MgCl_2_); and TRIS (2-Amino-2-(hydroxymethyl)propane-1, 3-diol; pH 7.0) were sourced from Sigma-Aldrich.

### DNA preparation

Upon receipt and without any further purification, the ssDNA oligonucleotide stocks were fully rehydrated in molecular biology-grade water. Their concentration was determined by absorbance at 260 nm using the manufacturer reported molar extinction coefficient. 1 mg of Free AF powder was rehydrated in annealing buffer (12 mM MgCl_2_, 10 mM NaCl, 5 mM TRIS, pH 7.0) and the concentration determined by absorbance at 647 nm using the manufacturer reported molar extinction coefficient of 270,000 cm^−1^M^−1^. Working dNTP stocks were created by diluting the supplied mixture to a final dNTP concentration of 50 *μ*M in molecular biology-grade water.

To assemble the DX-tile, dsDNA, and ssDNA substrates, the ssDNA oligonucleotides were added to annealing buffer in 1:1 stoichiometry with each strand at 5 *μ*M. Free AF and dNTP samples were diluted in annealing buffer from working stocks to a final dye concentration of 5 *μ*M and a final dNTP concentration of 5 *μ*M. Each structure was annealed in a thermocycler by heating the reaction mixture to 85 °C for 10 min, from 85 °C the temperature was decreased by 1 °C per minute until reaching 4 °C. Final sample concentrations were assumed to be 5 *μ*M (assembled structure) based on the 1:1 input stoichiometry. The utilized DX-tile design and formation was previously characterized via 10% polyacrylamide in 1X TRIS-Borate-EDTA (TBE) gel electrophoresis (PAGE) analysis [33].

### PIC dye stock preparation

200 *μ*M PIC dye stock solution was prepared from powder by dissolving in measurement buffer (10 mM NaCl, 5 mM TRIS, pH 7.0). To ensure fully solvated dye, the dye solution was sonicated for 1 h at 60 °C. The solution was then allowed to cool to room temperature and filtered through a 0.2 *μ*m syringe filter. The final PIC concentration was calculated by UV–vis absorbance using the PIC monomer’s molar extinction coefficient of 53,500 M^−1^ cm^−1^ at 523 nm.

### DNA-PIC sample preparation

Unless otherwise noted, all samples were prepared in measurement buffer by adding the necessary volumes of PIC stock and assembled DNA nanostructure solution to reach a final DNA nanostructure concentration of 0.5 *μ*M and a PIC dye concentration of 160 *μ*M (320-fold excess). J-bit samples were prepared by adding the necessary stock volumes to reach a DNA concentration of 0.5 *μ*M and a PIC concentration of 60 *μ*M (120-fold excess) [30, 33]. The spectra presented in the manuscript were collected approximately 36 h after sample preparation to allow for sufficient SA formation. Additional measurements were made 0, 12, and 24 h after sample preparation and selected spectra can be seen in the SI.

### Absorbance and fluorescence measurements

Absorbance and fluorescence emission spectra were captured using a TECAN Spark plate reader with samples loaded into a white Corning clear bottom 384 flat well plate (Product #3570) and each well containing 50 *μ*l of solution. To prevent liquid evaporation, the plate was covered with a UV-transparent sealable cover. Samples were measured at 20 °C with an *n* = 3 of replicates and the presented spectra are the average of the three. Absorbance scan spectra were collected from 300 to 800 nm. Fluorescence emission spectra were collected with a slit width of 5 nm and were measured from 660 to 800 nm using a 647 nm excitation, 595 to 800 nm using a 580 nm excitation, and 540 to 800 using a 523 nm excitation. Fluorescence excitation spectra were measured by collecting emission at 700 nm as a function of excitation wavelength with 5 nm slit widths. For further details regarding spectral processing and correction as well as the calculation of relevant photophysical dye parameters, see SI.

### Circular dichroism measurements

CD spectra were collected ranging from 200 to 700 nm with a J-1500 JASCO spectrometer. Each measurement was made at 20 °C, with the exception of the temperature dependent studies, and the presented spectra are the average of *n* = 3 repeats. Measurements were made using a 1 cm path length cuvette, unless noted otherwise. During overnight CD experiments, measurements were collected as outlined above every 8 min for 20 h summing to a total of 150 measurements.

### Atomic force microscopy visualization

AFM imaging was performed on a fast-scan AFM by JPK Instruments (Germany) under AC fast imaging mode (liquid) with USC-F0.3-k0.3 AFM tips from NanoWorld (Neuchâtel, Switzerland). On a segment of freshly cleaved mica (0.9 cm diameter; Ted Pella Inc.) mounted to a magnetic puck, 15 *μ*l of PIC-DNA solution was deposited immediately before measurement. A 25 *μ*l droplet of imaging buffer was deposited on the AFM tip, then the AFM tip mount was lowered into the sample buffer to create a liquid ‘chamber’ for imaging.

### Relative emission change

The relative change in PIC and AF emission (*R*_*c*_) was calculated in various conditions to estimate the SA’s ability to harvest light. The relative emission change is the percent increase or decrease of the integrated emission spectra in the presence of SA compared to the absence of SA. Hence, *R*_*c*_ is defined as:

(1) $$R_{c}=\left(\frac{\Psi_{s}-\Psi_{ref}}{\Psi_{ref}}\right)\times 100\text{\%}$$

where Ψ denotes the integrated emission, the subscript *s* denotes samples with SA present and the subscript *ref* denotes reference samples without the SA.

### Coherence length estimate

Based on the observations of Spano and Yamagata, an estimate of the *N*_coh_ can be obtained for linear aggregates from the fluorescence emission spectra [21].

(2) $$N_{coh}=\lambda^{2}*S_{R}$$

Where *S*_*R*_ is obtained experimentally from the 0–0 and 0–1 line strength ratio and λ^2^ is the Huang-Rhys factor. The λ^2^ can be estimated experimentally by equation 3, as described in Rolczynski *et al* [45].

(3) $$\lambda^{2}=\left(\frac{\gamma }{2*\Delta E_{v}}+0.25\right)$$

Where γ is the measured Stokes shift of the PIC structure and Δ*E*_*v*_ is the measured vibrational energy-level spacing.

## Results and discussion

### DNA nanostructure and sample design

The mixing of DNA and generic cyanine dyes has been known to produce helical dye aggregates since the late 1990s [38]. However, due to the non-covalent nature of these cyanine aggregates, elucidation of their molecular superstructure on a per-assembly basis is difficult and has been the subject of intense investigation [8]. One approach to further understand the complex relationship between the observed optical properties and underlying aggregate structure has been to use PIC dye as a model system [30–32]. In fact, the SA was initially discovered by our group, though not reported, investigating the limits of J-bit exciton relays [33]. We observed the appearance of a new redshifted absorbance peak in PIC-DNA samples that had partially evaporated during measurement, leading us to believe the effect was the result of increasing sample concentration. Furthermore, when collecting emission spectra, the concentrated samples appeared to outperform the J-bit samples in terms of energy transfer efficiency. This led us to prepare increasingly concentrated samples, and upon measuring their excitation spectra (figure S5), we discovered the previously unreported SA.

Crucial to the analysis of self-assembled DNA-based PIC aggregates is careful inspection of the DNA scaffold’s effect on the dye properties. For example, J-bits require an AT-track template of a minimal length (>8 bps) to form [30–33]. The versatility of DNA sequence design allows the AT-track to be positioned within a simple DNA duplex as well as more complex structures such as a DNA lattice [44, 46]. While the AT-track is presently the only J-bit requisite, the overall structural considerations are not trivial and differences in DNA superstructure are known to affect the optical properties of J-bits [30]. For example, J-bits formed with a duplex scaffold [31, 32] display features distinct from those formed with a DX-tile scaffold [30, 33]. Duplex bound J-bits result in a narrow red-shifted absorbance peak, but DX-tile bound J-bits result in the appearance of a broad shoulder appearing on the red side of the PIC monomer absorption band (523 nm) [30–33]. Therefore, to make characterization of the SA generalized, we utilized four simple, yet structurally distinct DNA oligonucleotide substrates.

**Figure 1.**
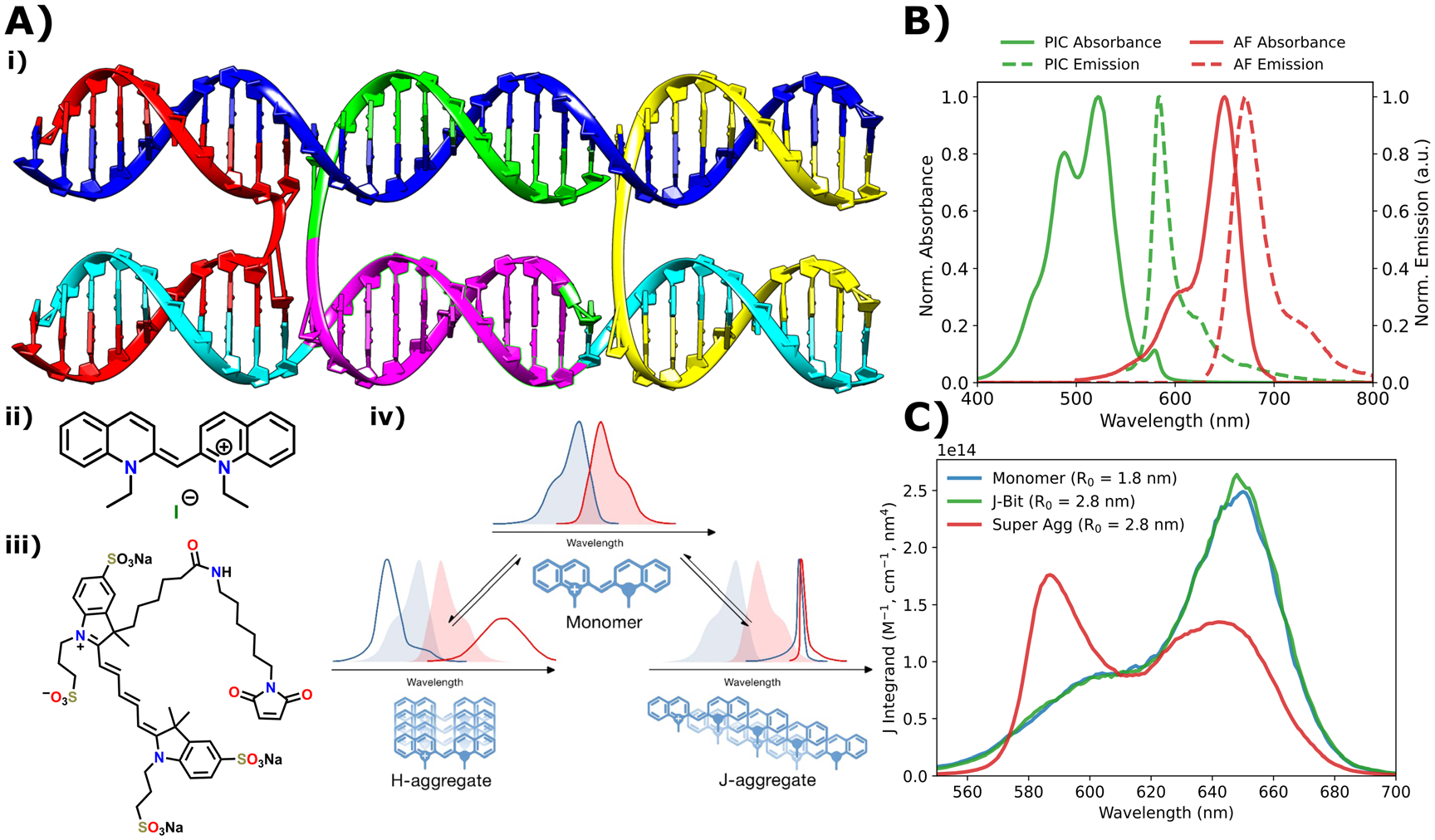
DNA nanostructure schematics and dye component properties. (**A**) (**i**) A molecular model representation of the DX-tile nanostructure self-assembled from five color coded component strands. The purple region denotes the AT- or GC-track regions. For full DNA strand sequences see the SI figures S1–4. (**ii**) Chemical structure of PIC dye. (**iii**) Chemical structure of AlexaFluor^™^ 647-maleimide. (**iv**) Schematic representation of structural models for cyanine dye H- and J-aggregation along with the characteristic spectral changes. These are provided as visual guides to the reader and do not necessarily represent the SA structures. Panel modified from [49]. (**B**) Normalized absorbance (solid) and emission (dashed) spectra of SA (green) and AF (red) dye components. For monomer and J-bit spectra, see the SI. (**C**) Spectral overlap integrand plotted as a function of wavelength for the donor species PIC monomer (blue), J-bit (green), and SA (red) and an AF acceptor dye. The estimated *R*_0_ values are reported in order to differentiate between the expected transfer efficiency of the monomer and J-bit species. Reproduced from [49]. © IOP Publishing Ltd. All rights reserved.

The first two structures were DX-tiles assembled from five ssDNA strands (figure 1(A)). In general, a DX-tile can be described as a mechanically rigid structural unit used to form larger scale nucleic acid lattices [46, 47]. In the context of the DNA nanotechnology field, DX-tiles allow for the straightforward integration of multifunctional materials since the base structural unit is inherently modular and easily modifiable. While there are multiple DNA nanostructures that have this property, the advantages of the DX-tile lie in its design tractability and the capacity to co-localize two parallel DNA duplex helices, in which the sequence can be fine-tuned to position AT-tracks very precisely [30, 33]. DX-tiles achieve this through the two antiparallel Holliday junction motifs, which constrain displacement of the helices [48]. In the context of our particular scaffolds, the principal difference between the two DX-tiles was that one contained a 10 bp AT-track, hence this structure is referred to as AT. The other DX-tile contained a 10 bp alternating poly(GC) sequence (GC-track) replacing the AT-track, hence this structure was referred to as GC. We include the GC structure because GC-tracks have been observed to disrupt PIC J-bit formation and attenuate the associated coherent properties [31–33]. While the core structure remains the same for both GC and AT nanostructures, we modified the sequence of the green and cyan strands (figure 1(A)) to include the AT- or GC-track, specifically limited to the region where they overlap highlighted in purple (see SI for sequences and schematics). Structures which included an AF reporter incorporated a labeled strand (yellow), to which an AF dye was tethered through a six-carbon linker (Figure S4). Thermal annealing and structural fidelity were previously reported and characterized with estimated > 90% correct formation efficiency [33]. The dsDNA (duplex) structures were designed by taking the dye labeled strand (yellow) and annealing it with a modified blue strand such that it was fully complementary with the yellow strand (figure 1(A)). Finally, ssDNA samples were simple mixtures of the dye labeled strand. It is important to note that the dsDNA and ssDNA scaffolds do not include AT- nor GC-tracks within the sequence and have a relatively uniformly distributed GC content of ~60%.

When mixed together, PIC has repeatedly demonstrated an affinity to bind to DNA. The cationic nature of PIC and the anionic nature of the nucleic acid phosphate backbone tend to promote electrostatic binding between the two molecules [50, 51]. Molecular dynamics simulations support this, indicating that PIC likely binds in the minor groove of the DNA where the phosphate charges are readily accessible and steric hindrance is not as limiting compared to intercalation directly between the nitrogenous base sequence [22, 30]. Though the exact mechanism is still under debate, electrostatically driven interactions are supported experimentally since it has been shown that even GC-track controls, which theoretically prevent J-bit formation, still exhibit significant non-specific PIC binding to DNA scaffolds [32, 33]. Furthermore, substantially changing the ionic strength of the buffer has shown to disrupt formation [30]. However, when PIC is mixed with AT-track DNA, PIC dye’s preference for AT bps leads to dense packing where Van der Waals forces can direct interactions and promote stronger excitonic coupling [30–33]. Since PIC aggregation is a non-covalent process, the relative concentrations of dye and scaffold play an important role and coherent PIC aggregates are only able form if the concentration ratio of PIC to DNA is above a critical proportion. Boulais *et al* found that a concentration ratio of 90 *μ*M PIC monomer and 75 *μ*M total dA·dT bases induced optimal J-aggregation. Using isothermal titration calorimetry, they reported PIC-AT-track dissociation constants of 20 to 25 *μ*M for duplex DNA and 66 *μ*M for DX-tile templates. Spectroscopic experiments were executed using a PIC dye concentration of 90 *μ*M and a fully formed DX-tile concentration of 0.72 *μ*M (120-fold excess) [30]. This finding was supported by further investigation by our group where diminishing CD peak intensity was observed at higher PIC concentration, indicating binding site saturation at concentrations in line with this dissociation constant. This investigation utilized an optimal ratio of 52 *μ*M PIC and 0.4 *μ*M DX-tile (130-fold excess) [33]. To our knowledge these are the only two examples of PIC aggregates formed on DNA templates more complex than simple duplex DNA and engaging in energy transfer. Both Mandal *et al* and Banal *et al* successfully formed J-bits in DNA duplex templates using a lower ratio of 90 *μ*M PIC dye and between 2 and 5 *μ*M DNA, in line with the lower predicted dissociation constant for binding to duplex structures [31, 32].

However, in preliminary experiments, when the ratio of PIC to DX-tile was elevated to at least 320-fold, distinct changes in the corresponding optical spectra were observed. All reported experiments with SA samples were formed using this elevated (320-fold) excess. The normalized absorbance and emission spectra of the SA and AF are displayed in figure 1(B). Accompanying the optical spectra are the spectral overlaps of PIC monomer (blue), J-bit (green) and SA (red) with AF plotted as a function of wavelength (figure 1(C)). The monomer and J-bit have nearly identical spectral overlap profiles with the AF, due to PIC’s broad emission profile. These two species however can be distinguished by their Förster radii (*R*_0_) where the J-bit is nearly double that of the monomer due to the order of magnitude greater quantum yield (QY) of the J-bit [31]. The SA on the other hand displays a shift in overlap to between 580 and 600 nm due to corresponding emission bandwidth narrowing (discussed below), though the *R*_0_ coincidentally has the same value as the J-bit.

### Absorbance characterization

**Figure 2.**
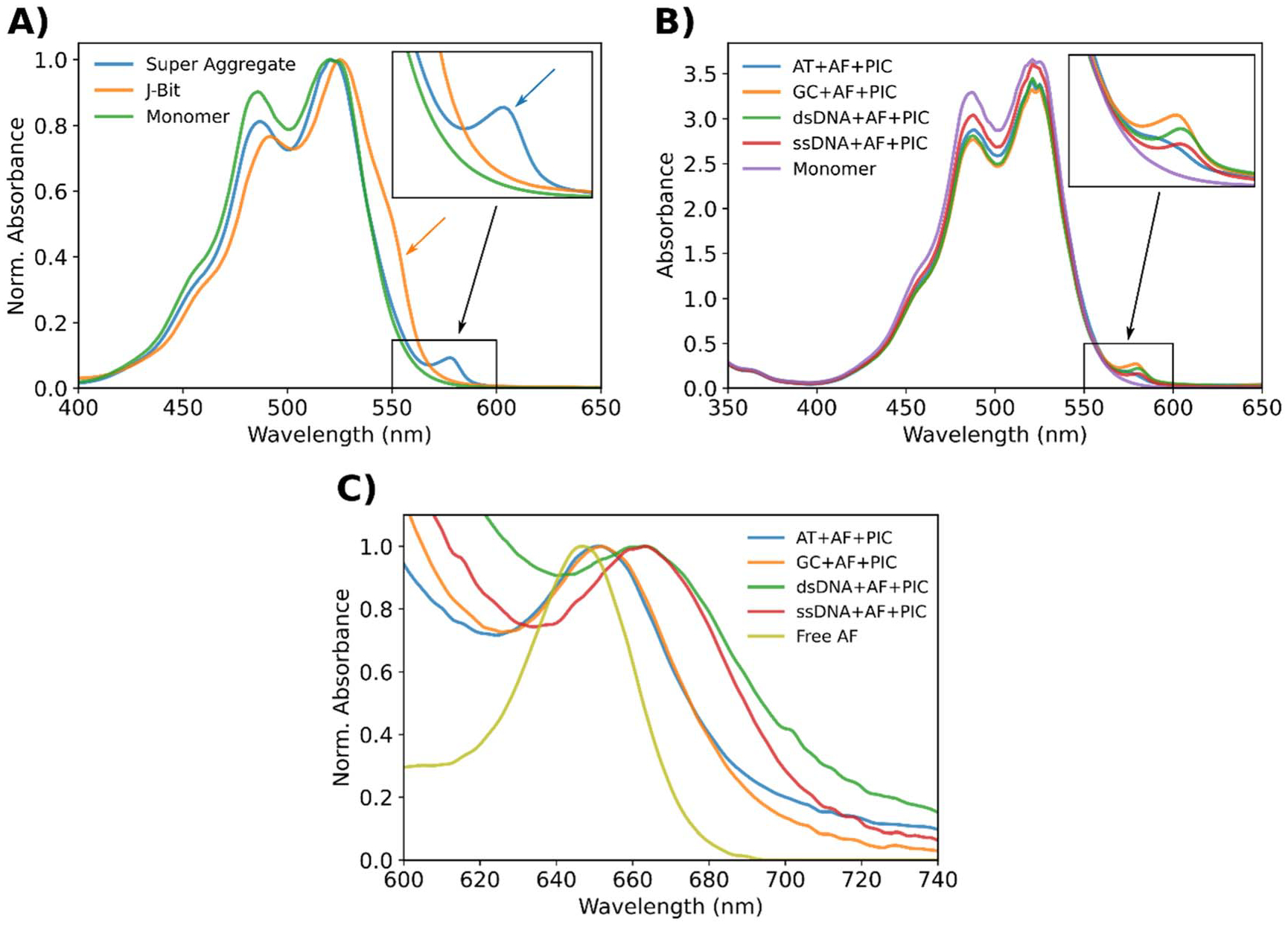
Absorbance features characteristic of SA formation. Except for the J-bit, each of these samples were formed by mixing 160 *μ*M PIC dye with 500 nM DNA normalized to the dye labeled strand concentration. The J-bit sample was formed in the AT nanostructure by mixing with 60 *μ*M PIC dye. (**A**) Normalized absorbance spectra of PIC SA (blue), J-bit (orange), and monomer (green). The color-coded arrows highlight the characteristic features which differentiate J-bit (555 nm shoulder) from the SA (580 nm peak). Inset is a magnification of the black box region. (**B**) Absorbance spectra of SA formed in an AT (blue), GC (orange), dsDNA (green), and ssDNA (red) nanostructures. The SA absorbances are presented relative to a PIC only control (purple), which is considered representative of a monomeric population. Inset is a magnification of the black box region. (**C**) Normalized absorbance spectra of DNA + AF + PIC samples with spectra centered on the AF absorbance band. The color-coded curves are presented relative to the Free AF control which is to highlight the red shifting and linewidth broadening.

PIC SAs can be easily identified by their distinctive absorbance spectra which distinguishes them from both PIC monomer and DNA bound J-bits. The SA display an emergent absorbance peak located at ~580 nm highlighted by the black arrow in figures 2(A) and (B). For reference, the PIC monomer can be identified by absorbance peaks at approximately ~490 and ~520 nm. While J-bits templated in DX-tile scaffolds typically manifest as an absorbance shoulder located on the red side of the monomer 0–0 absorption band (~550 nm) (figure 2(A) orange arrow) and is easily distinguishable from the SA (figure 2(A) blue arrow) in experimental spectra. The SA absorbance peak appeared in both DNA + AF + PIC (figure 2(A)) and DNA + PIC (figure S7) samples suggesting the feature is more than the result of simple dye-dye interactions driven by hydrophobicity or bulk dye concentration. Controls lacking DNA oligonucleotides (i.e. Free AF + PIC, dNTP + PIC and PIC only) displayed no change from baseline in the characteristic SA absorbance region (figure S8). This suggests some critical characteristic of the polynucleotide sequence is essential for the formation of PIC SAs.

In addition to the emergent absorbance peak, we also identified features which suggest the SA affects the energy levels of the AF. Controls lacking PIC (DNA + AF) exhibited extinction coefficient and spectral linewidths consistent with the Free AF dye (figure S10). The characteristics of the Free AF dye in solution were consistent when mixed with and without PIC (Free AF versus Free AF + PIC) (figure S9). Finally, the PIC only controls displayed no evidence of peak formation in this region either (figure S11). These results indicate neither the bulk dye nor the DNA scaffold alone are directly affecting the AF transitions. However, comparing AF labeled nanostructures which form SAs (DNA + AF + PIC) to the Free AF control, we observed significant decreases in extinction coefficient, absorption peak redshifts, and linewidth broadening (figures 2(C) and S12–S13). Collectively this data is strongly suggestive that the changes were a result of local interactions between the SA and AF with a clear distinction between DX-tile samples (AT and GC) and non-DX-tiles samples (dsDNA and ssDNA). For example, we can observe the AF S0 to S1 transition energy appears to be modified by the presence of SA. This is evident by the redshifting of the AF absorption peak in DNA + AF + PIC samples relative to the Free AF control (figure 2(C)). While the Free AF absorbance peak was in line with manufacturer specifications (647 nm) the DX-tile samples exhibited 5 nm (14 meV) redshifts to 652 nm while the non-DX-tile samples exhibited 17 nm (49 meV) redshifts to 664 nm. The latter corresponds to a 2.5% decrease in the S0-S1 transition energy of the AF.

Since absorption readings are ensemble measurements, the observed spectra are the summed result from the contributions of molecular subpopulations. If the heterogeneity or relative contribution of these subpopulations is unknown it can preclude certain detailed analyses. The non-covalent nature of the PIC aggregates means more degrees of freedom and thus access to more available states, ultimately reducing resolution (spectral broadening). This fact combined with the large bulk dye excess prevented us from explicitly determining the concentration or proportion of PIC dye forming aggregates. However, the photophysical properties strongly imply we are nucleating a new form of PIC aggregate (the SA) which is easily discernable from both J-bit and monomeric PIC. From the results of negative controls, we believe the formation of this SA requires a DNA oligonucleotide substrate. Finally, from the changes in AF absorbance, we interpreted the data as evidence implying the SA is directly interacting with the AF dye. Therefore, we speculate that during SA formation the AF dye is being integrated into the aggregate and therefore strongly coupling with the PIC dyes in the SA. Additionally, since Free AF added to the concentrated PIC solution (figure S9) resulted in no spectral changes, it further supports the theory SA is formed on the basis of the DNA. Furthermore, the characteristics of this coupling strongly depend on the DNA substrate design.

### Changes in PIC emission

**Figure 3.**
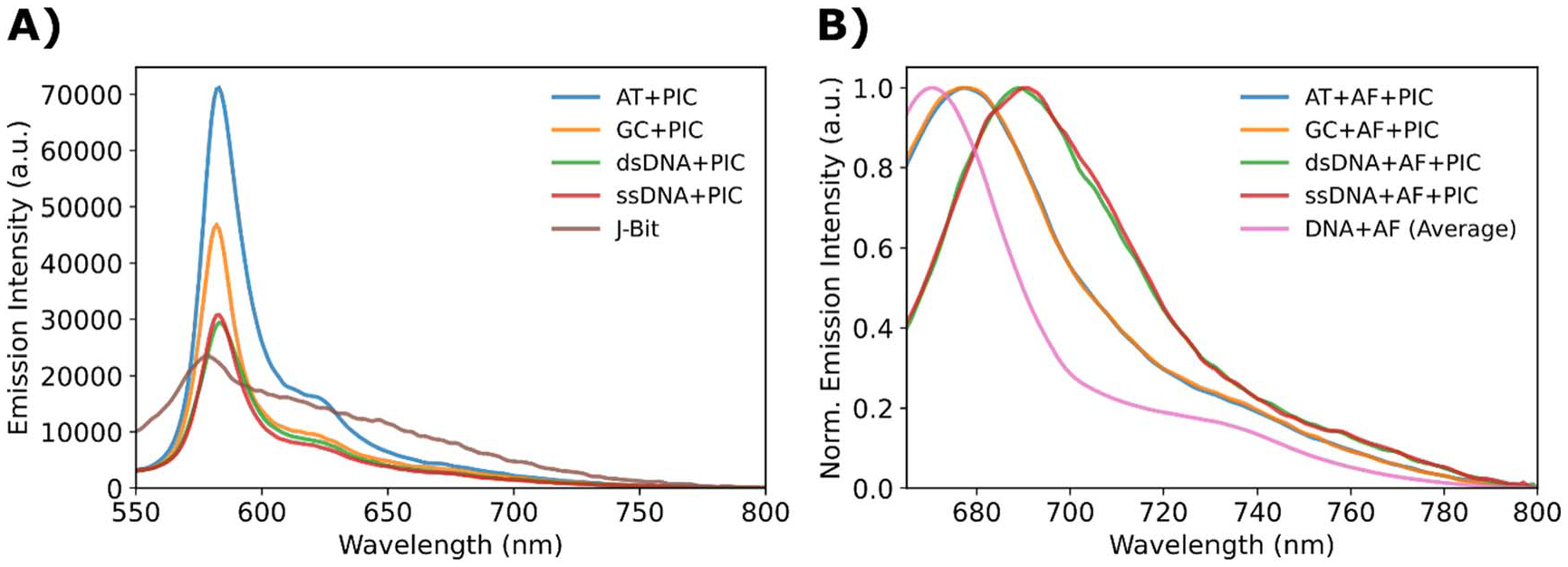
Fluorescence emission spectra of directly exciting dye components. (A) Fluorescence emission spectra of DNA + PIC samples in response to 523 nm excitation. The color-coded curves are presented relative to the emission of J-bit formed in the AT nanostructure (brown). (**B**) Normalized fluorescence emission spectra of DNA + AF + PIC samples in response to 647 nm excitation. The color-coded curves are presented compared to the non-SA (DNA + AF) controls of the four DNA substrates averaged together.

Fluorescence emission spectra were also collected and significant changes in the emission of both PIC and AF dye components was observed. Displayed in figure 3(A) are fluorescence emission spectra utilizing a 523 nm excitation wavelength of the different PIC-DNA mixtures. The 523 nm excitation was used to directly probe the photophysical properties of the SA. This excitation wavelength corresponds to the PIC dye’s maximum absorption and has been used previously to excite both PIC monomer and J-bit [30–33]. The 523 nm excitation is advantageous because it allows for collection of the entire SA emission spectrum. The small Stokes shift between the SA absorption (~580 nm) and emission (~582 nm) maximum, though supportive of the SA’s J-aggregate like nature, typically leads to bleed through of the excitation beam in the regions closest to the excitation wavelength. The SA samples (DNA + PIC) were formed using the typical SA concentration ratio (320-fold excess PIC) and presented in comparison to the J-bit, which was formed using the AT nanostructure as a scaffold with a 120-fold PIC to DNA concentration ratio. To obtain the final spectra as seen in figure 3(A), the J-bit spectrum was linearly corrected to compensate for the difference in concentration (see SI). Comparison to the J-bit allows for a relatively well characterized reference by which we can measure changes in the SA photophysics.

Qualitatively, SA samples exhibited significant linewidth narrowing and a stronger PIC emission compared to the J-bit. The SA emission also exhibited the appearance of red shoulder characteristic of other cyanine dye emissions [45, 52, 53]. Typically, linewidth narrowing is correlated with more homogenously-structured aggregates [54]. The J-bit emission on the other hand is much weaker, but its broader width makes it difficult to determine which samples have the greater total photon emission. Therefore, we analyzed the integrated emission spectra to calculate the relative changes in emission (*R*_*c*_) as well as estimate the QY (Φ), the values are distinct due to the subtle changes in the absorbance spectra which are not considered for the *R*_*c*_ but are considered for Φ. Each SA QY estimate is calculated utilizing the J-bit as a secondary standard (Φ = 0.18 ± 0.03%) [30]. Interestingly, although each of the samples exhibited significantly narrowed emission peaks, only the AT structure had an increase in emission relative to J-bit (*R*_*c*_ = 15.4 ± 1.5%). Though the GC structure resulted in decreased emission (*R*_*c*_ = −25.7 ± 0.6%), both DX-tiles emitted significantly more photons than either of the non-DX-tile oligonucleotides. The dsDNA and ssDNA resulted in emission decreases of −40.8 ± 1.1% and −43.7 ± 0.6% respectively (table 1). The PIC only control, which is considered a monomer, displayed a > 70% decrease in emission, in line with reported literature values [30–33].

Although slightly increased emission was observed in the AT sample, the uncertainties are such that it does not translate to a statistically significant change in QY between the AT (Φ = 0.23 ± 0.3%) and J-bit reference. The GC sample (Φ = 0.15 ± 0.2%), displayed a slight decrease in photon emission but considering uncertainty, this again does not translate to a statistically significant change. However, for the other samples, significant decreases in QY were observed. This phenomenon is not uncommon for strongly coupled cyanine dyes which have been observed to exhibit QY quenching [10, 34, 45, 52, 55]. While the exact mechanism behind this emission quenching is uncertain, it is probable that new relaxation pathways open due to the close interactions. This aspect is discussed further below in the Energy Transfer Characterization section.

The fluorescence emission spectra can provide additional information as detailed in Spano and Yamagata [21]. The ratio of the (0–0) and (0–1) peaks (~584 and 626 nm, respectively), in conjunction with λ^2^ can provide an estimate of *N*_coh_. References [21] and [45] supply details, with equations 2 and 3 in the Methods section providing the means to obtain the estimates. From the representative spectra in figure 3(A), taking into account background and peak broadening we obtain *S*_*R*_ of 31.1 ± 4.4 for the AT and GC samples and 19.8 ± 2.2 for the dsDNA and ssDNA. The J-bit and PIC had *S*_*R*_ of 2.3 ± 0.6 and 1.5 ± 0.4, respectively. Estimates for λ^2^ resulted in an average value of 0.34 for all four SAs, while the J-bit λ^2^ was 0.62. These values are in line with those reported for other DNA conjugated cyanine dyes [45]. The approximations ultimately cumulate in the SA DX-tile structures having an *N*_coh_ = 10.2 ± 1.5, while the more flexible dsDNA and ssDNA had *N*_coh_ = 6.7 ± 0.8. Both values for the SA are longer than the 1.6 ± 0.4 of the J-bit (monomeric PIC had *N*_coh_ < 1 as would be expected), thus illustrating the stronger interaction of the SA aggregate, observed in the sharper emission band as well. We highlight the *N*_coh_ of 1.6 is in line with the *N*_coh_ ~2 reported for other experimentally measured J-bit structures in the literature [30, 31], which adds support to the experimental approach.

### Changes in AF emission

In addition to monitoring changes in PIC dye, we also examined how the photophysical properties of the AF may be modified by SA formation. Here, SA was formed with AF labeled DNA scaffolds (DNA + AF + PIC) and was compared to controls lacking PIC (DNA + AF). To directly probe the AF, each sample set was excited using a 647 nm wavelength. The 647 nm excitation allows for optimal excitation of the AF dye while also being an absorption minimum for PIC dye (figure S11). This is confirmed by the lack of inner filter observed when comparing the emission spectra of the Free AF *versus* Free AF + PIC samples (figure S14).

The most striking change in AF can be observed in the calculated values of *R*_*c*_ (table 1), where each of the nanostructures exhibit significant emission quenching. The AT and GC structures had −80.8 ± 2.6% and −84.6 ± 1.4% decreases in emission respectively, while the dsDNA and ssDNA samples displayed greater quenching with −95.5 ± 0.2% and −95.9 ± 0.3% decreases. Additionally, when the emission spectra were normalized, substantial redshifting of the AF emission was observed (figure 3(B)). The DNA + AF controls displayed an emission maximum wavelength on average of 671 nm. When in the presence of SA, the DX-tile samples exhibited 7 nm redshifts (16 meV), while the non-DX-tile samples exhibited 20 nm shifts (50 meV), and all peaks appear broader relative to DNA + AF controls. Finally, even though the timescale of carrier recombination should allow for solvent reorientation to affect the emission wavelength [56], based on the absence of peak shifts in the Free AF versus Free AF + PIC controls (figure S14), we conclude the bulk PIC is not contributing through this mechanism.

AF QY estimations were also calculated based on the AT + AF as a secondary standard (Φ = 41.2 ± 2.6%) [33]. The significant reductions in emission led to quenched QY values with the DX-tiles showing Φ = 12.8 ± 4.6% and 12.1 ± 2.5% for the AT and GC constructs respectively. The non-DX-tile structures exhibited a near order of magnitude decrease in QY with values of 4.8 ± 0.9% and 6.1 ± 3.6% for the dsDNA and ssDNA respectively. We interpret these results, collectively with the shifts in absorbance spectra discussed above, as evidence of strong coupling between the AF dye and the PIC SA. However, the consistent similarities between the DX-tile versus the non-DX-tile samples implies at least two distinct aggregate configurations and emphasizes the role of the DNA scaffold in controlling aggregate properties.

### Energy transfer characterization

**Figure 4.**
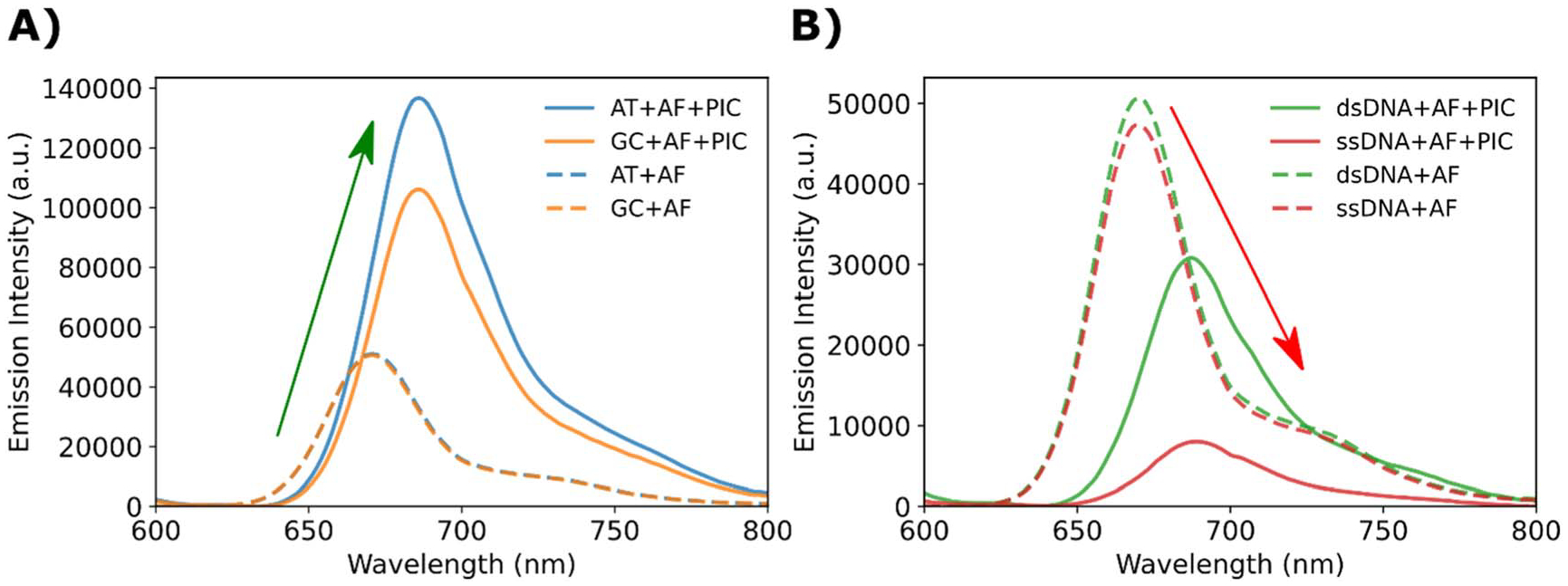
SA excitation in the presence of AF and subsequent energy transfer. (**A**) Fluorescence emission spectra of DX-tile (DNA + AF + PIC) samples in response to 580 nm excitation. The color-coded curves are presented relative to the corresponding PIC lacking controls (DNA + AF). (**B**) Fluorescence emission spectra of non-DX-tile (DNA + AF + PIC) samples in response to 580 nm excitation. The color-coded curves are presented relative to the corresponding PIC lacking controls (DNA + AF).

A motivating interest of DNA-based coherently coupled aggregates is to rationally control energy flow on the nanoscale with high efficiency [6–8, 49]. This means in addition to controlling the innate properties of aggregates, they must necessarily engage in predictable energy transfer. To determine the potential energy transfer properties associated with SA formation, we utilized the same sample set as was used to probe the AF component properties (*i.e*., DNA + AF + PIC *versus* DNA + AF). These two datasets were each excited using a 580 nm wavelength which is optimal for SA excitation and the resulting spectra are displayed in figure 4.

The DX-tile-based SA samples (figure 4(A)) resulted in sensitized AF emission while the non-DX-tile-based SA samples (figure 4(B)) resulted in AF emission quenching. Table 2 quantifies the relative changes in emission, and we can see that the AT induces greater sensitization (*R*_*c*_ = 195 ± 2%) than GC (*R*_*c*_ = 131 ± 2%), while the ssDNA sample (*R*_*c*_ = −81.4 ± 0.1%) is quenched more than the dsDNA sample (*R*_*c*_ = −33.3 ± 0.2%). In contrast to the changes in relative emission, both the DX- and non-DX-tile samples exhibit approximately equal emission redshifts of about 15 nm.

and appear to have similarly broadened emission (figure 4). This illustrates a clear difference from what was seen in figure 3(B), where the dsDNA and ssDNA with SA had considerably more red-shifted AF peaks than the DX-tile SA samples.

Though the previously discussed features in emission spectra suggest strong coupling is occurring, the relative differences between DX-tile versus non-DX-tile samples strongly support the hypothesis that there exists a difference in the underlying dye configuration. If we consider the uncertainties of the AF QY measurements, we can postulate two AF configurations in the presence of SA. The DX-tile systems (both AT and GC) add sufficient rigidity such that the QY of the AF can be reported as 12 ± 4%. In the case of the more dynamic dsDNA and ssDNA, the values are statistically inseparable and can be reported as 6 ± 3%. This suggests the AF is brighter in the DX-tiles as well as coupled to a larger antenna (greater *N*_coh_) than the dsDNA and ssDNA samples, consistent with the greater *R*_c_ of the DX-tile samples. We hypothesize the decreased rigidity of the dsDNA and ssDNA considerably diminishes the AF emission. In conjunction with the changes in absorbance spectra as well as the observed emission peak shifts of the AF, the decrease in QY strongly hints at the AF being integrated into the PIC aggregates. This integration, which results in strong coupling, inherently changes the dye’s energy levels and potentially opens new non-radiative decay pathways, as has been observed with other cyanine dyes [10, 34, 52, 53, 57]. These additional decay pathways are often correlated to rotation over the methylene bridge and as such, the added rigidity of DX-tiles likely plays a strong role in inhibiting this rotation, a phenomenon observed when these systems are studied under cryogenic conditions [52, 53, 58].

We were also particularly interested in the SA’s ability to act as an antenna, or its ability to efficiently harvest photons and funnel them to a collection site [59, 60]. Due to the dense packing and strong coupling, the absorbance cross-section of the SA should be considerable. To investigate the SAs performance as an antenna, we integrated the collected emission spectra and consider the AF as an energy acceptor. One method of measuring the antenna gain at a single wavelength of a specific system is by using the relative fluorescence change, *R*_C_, described in equation 1 (see Materials and Methods). The *R*_C_ can be expanded to understand the full system’s ability to transfer energy from the initial site of excitation to the final acceptor. For the latter, the total emission (Ψ), can be separated into components and represented in the following equations:

(4) $$\Psi_{S}=\sigma_{AF}\times \Phi_{AF}^{\prime }+\sigma_{PIC}\times ET\times \Phi_{AF}^{\prime }$$

(5) $$\Psi_{R}=\sigma_{AF}\times \Phi_{AF}$$

In these equations, *S* denotes the SA sample and *R* denotes the reference sample lacking SA, *σ* represents the component’s absorbance cross-section, Φ is the AF QY while, Φ′ recognizes that the AF QY is modified upon SA formation as discussed above. This modification accounts for the decrease in QY, and the fact that Φ′ is distinct for the DX-tile and non-DX samples. The energy transfer efficiency, ET, represents the SA’s ability to transfer excitons to the AF. We employ ET as a general metric as we are unable to illuminate a particular mechanism. We speculate a strongly coupled coherent transfer mechanism dominates, yet it is likely Förster as well as Dexter mechanisms may play a part as well. This is supported by previous studies of DNA-based PIC aggregates which suggested coherence lengths of 2–5 dyes, significantly shorter than the total size of the aggregate (up to 12 molecules per turn of B-form DNA) and the total length of energy transfer (greater than 10 nm) [30, 31]. In these cases, it is evident that Förster and/or Dexter mechanisms must play a role. In the case of SA samples, the absorbance values at 580 nm are good estimates of *σ*; using the measured AF QY (Φ = 0.41) and SA modified QY values (Φ′_*DX*_ = 0.12 ± 0.04 and Φ′_*non*-*DX*_ = 0.06 ± 0.03) we can obtain projections for the ET (table 2). The specific ET values provide important insight into the mobility of excitons within the SA and their subsequent transfer to the AF. The clear result is that the energy shuttling properties (from PIC molecule to PIC molecule) are very distinct depending on the DNA substrate which was used to initiate the aggregation as seen in the different *N*_coh_ values. As the literature has shown previously, the existence of AT-track DNA results in more ordered J-like structures and thus it is not surprising this system displayed the most efficient energy shuttling and ET [30–33]. Comparatively, the GC and dsDNA samples display 3-fold decreases in ET, as compared to AT, but are statistically inseparable. The difference in emission but agreement in ET strongly supports our hypothesis that the DNA substrate drives nucleation of the SA and can cause the PIC molecules within the aggregate to adopt distinct conformations. These conformations result in unique energy shuttling and energy transfer properties and as such, their function as antennae is also unique. The GC has a greater *R*_c_ as well as *N*_coh_, yet the calculated ET is the same as the dsDNA, alluding to the coupling mechanism from the PIC to the AF as being very similar. Finally, the ssDNA sample displays an 8-fold decrease relative to the AT, exhibiting the lowest degree of ET likely due to its significantly increased flexibility and structural disorder [61, 62]. Collectively this data indicates that rigidity in addition to the sequence of the DNA play an important role in defining the photonic properties of PIC aggregates and should not be overlooked. The interaction between the DNA template and SA also plays a role in the integration of secondary dyes, for example the AF functioning as an acceptor in this case [41]. Subsequent CD and AFM characterization (*vide infra*) were implemented to find support for this hypothesis.

### Circular dichroism characterization

Additional characterization was undertaken using CD spectroscopy which is the chiroptical counterpart to UV–vis absorption spectroscopy. CD spectrophotometers are sensitive to the absolute configuration of a sample in correspondence to their electronic transitions. Generally speaking, the advantage of CD spectroscopy is twofold. Firstly, the DNA B-form alpha helix which forms spontaneously in aqueous buffer has inherent right-handed chirality [2, 63]. Therefore, the DNA nanostructures and often the molecular species associated with the DNA helix exhibit a signal in a CD spectrum [64]. Secondly, when two or more chromophores are strongly coupled, if the TDMs are not exactly coplanar or collinear, the electronic structure of the aggregate is skewed and produces a CD spectrum [65, 66]. Thus, when interacting with EM radiation, two strongly coupled chromophores can, depending on the TDM alignment, respond in-phase or anti-phase. In these cases, the skewed electronic structure results in helical charge displacement, generally known as an exciton couplet [67]. PIC aggregates and crucially J-bits have been demonstrated to act in this way. Thus, CD spectra have been extensively used to identify and characterize strongly coupled PIC aggregates in solution and on DNA substrates [22, 30–33, 50, 51].

**Figure 5.**
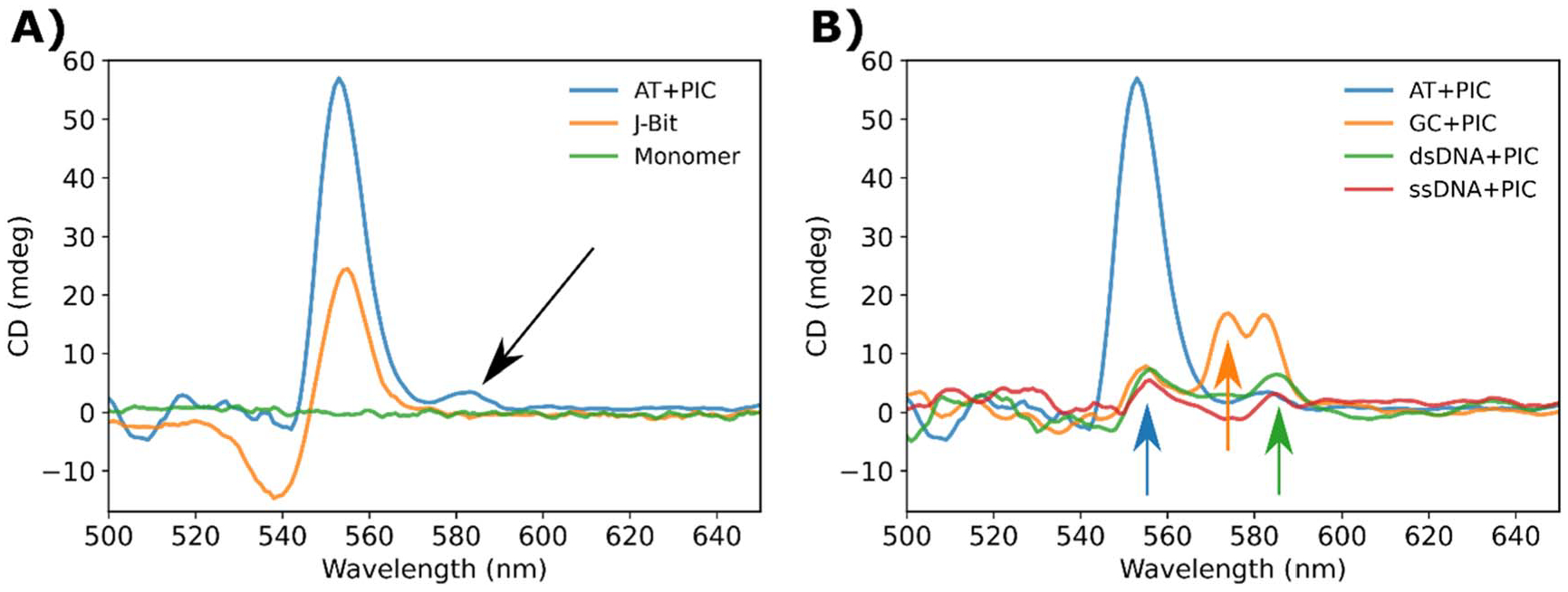
CD spectra of SA. (A) CD spectra of PIC SA (blue), J-bit (orange), and monomer (green). The black arrow highlights an emergent positive peak located at approximately 580 nm. (B) CD spectra of SA formed in an AT (blue), GC (orange), dsDNA (green), and ssDNA (red) nanostructures.

Neither the DNA alone, nor PIC monomer in solution (figure 5(A) green) reveal any CD signal in the region of SA absorbance. However, we can contrast this with a typical J-bit CD signal (figure 5(A) orange), where we observe characteristic spectral features. The signal is characterized by an intense positive peak in the long-wavelength component and a weaker negative peak in the short-wavelength component. The CD signal crosses the baseline at the wavelength overlapping with the J-bit shoulder peak (~550 nm) and is characteristic of exciton coupling [30–33]. When drawing comparisons to the SA spectrum (figure 5(A) blue), we observe an increase in positive peak intensity approximately overlapping the J-bit region. This is not surprising because, as discussed above, we would typically expect J-bits to template in the AT-track regions of DNA which is corroborated by our fluorescence emission and ET measurements. Though the SA lacks the characteristic negative peak component at shorter wavelengths, this is not uncommon in strongly-coupled cyanine dye systems [55]. A more interesting aspect of the SA CD spectrum is the appearance of a feature located at approximately ~580 nm (figure 5(A) black arrow) nearly overlapping the SA peak in linear absorption spectra. Temperature dependent studies of the CD and absorbance spectra (See figure S15) showed that the 580 nm peak had a change in intensity depending on the solution temperature. Peak intensity was found around 15 °C with the peak almost entirely gone by 45 °C. In contrast, the J-bit peak was much less susceptible to temperature changes, peak intensity at 5 °C and still observed at 50 °C.

The AT + PIC SA was also compared with the other SA forming samples (figure 5(B)). All SA spectra retain some similarity as there seems to be a weak positive peak forming in the J-bit region, highlighted by the blue arrow. The weakness of the peak is unsurprising in that each of these samples lacks the AT-track requisite for efficient J-bit formation. Even though in theory, the DNA lacks the proper template sequence, previous studies have observed PIC nonspecifically interacting with both poly(GC) and poly(ATAT) sequences [30–33]. Since PIC is clearly prone to spontaneous aggregation, and the SA studies were conducted at significantly higher concentration regime, it is possible this allowed a small fraction of molecules to orient optimally and resulted in a subpopulation of J-bits. However, the difference in signal intensity clearly suggests this is a smaller fraction. In addition to the 555 nm peak, we also observed the presence of two new, positive, long-wavelength components at ~575 nm and ~585 nm highlighted by the green and red arrows respectively. In the GC sample, the two features are prominently displayed as two peaks (575 and 580 nm) which appear to be partially overlapping. Similar to what was seen in the AT sample, the peaks appear to be temperature dependent with their max intensity observed at 15 °C (see figure S15). In comparison the non-DX-tile samples only show a weak positive signal at ~585 nm. The unique signatures of the CD spectra partially correlate with the ET values shown in table 2. The 585 nm CD peak seen in both the GC and dsDNA suggests a similar SA structure, especially when contrasted with the quite distinct spectra of the AT samples, which resulted in the ~0.24 efficiency value for ET in the GC and dsDNA samples. The ds and ssDNA SA positive peaks are also slightly redshifted relative to the DX-tile SA peaks, further supporting the hypothesis that rigidity of the DNA substrate plays a crucial role in determining the underlying structure of the resulting aggregate. This phenomenon has been observed in other cyanine dyes such as Cy5 and Cy5.5 hetero- and homo-aggregates tethered to DNA Holliday junctions [55]. We note that the PIC aggregate’s CD spectra were non-conservative, i.e. the integral of the CD signal was non-zero. This is consistent with previously reported PIC CD spectra [22, 30, 33] and may arise from the heterogeneous nature of the samples (the mix of monomers and SA) as well as mixed transition states that might arise within the aggregates [68, 69].

### Atomic force microscopy visualization

The final characterization technique we deployed was AFM visualization. AFM is a scanning probe microscope technique which allows for the surface topography of a sample to be visualized with nm resolution. To achieve high resolution imaging of nucleic acid nanostructures, the DNA is often deposited onto a mica substrate, where mica electrostatically binds the DNA. Once deposited onto the mica, the imaging can be done in a hydrated environment as there is no additional required dehydration or staining of the DNA, a particularly convenient advantage of AFM.

Previous work examining aqueous PIC solutions identified spontaneously formed J-aggregates at mica/water interfaces [70–72]. The absorption and fluorescence properties of these aggregates were in line with the SA characteristics reported above. However, the formation of these solution-based aggregates is not in parity with the conditions for our SA formation. For instance, to form the J-aggregates, aqueous PIC solutions ranging from 0.1 to 4.0 mM were utilized for deposition [70]. In comparison, optimal SA formation using a DNA substrate was achieved with 0.16 mM PIC stock overlapping only with the low end of this previously tested range. At equivalent concentrations, solution-based J-aggregates formed leafy islands extending 400–600 nm in length and 80–100 nm in width. The number of leafy islands observed increased in correspondence with increasing the bulk PIC concentration [70, 71]. The observed islands were all 3–6 nm thick indicating the formation of at least several molecular layers and led the experimentalists to propose Volmer-Weber type growth process where discreet islands form and then coalesce into larger monolayers [70]. Interestingly, the thickness of the aggregates appeared to be independent of the PIC concentration during deposition, suggesting that the energy of adsorption to the mica is such that the aggregate is unstable at heights greater than 3–6 nm. At this distance from the surface, the PIC molecules would dissolve as the solubility of the dye overcomes the inclination to aggregate [70, 71]. However, these studies still acknowledge there may be fundamental differences between the J-aggregates formed in solution, which are proposed to be fiber- or ribbon-like, and those formed at the solution/mica interface. Below we report the observation of both linear PIC fibers and leafy islands, as well as larger crystalline islands which display distinct morphologies dependent on the DNA substrate.

**Figure 6.**
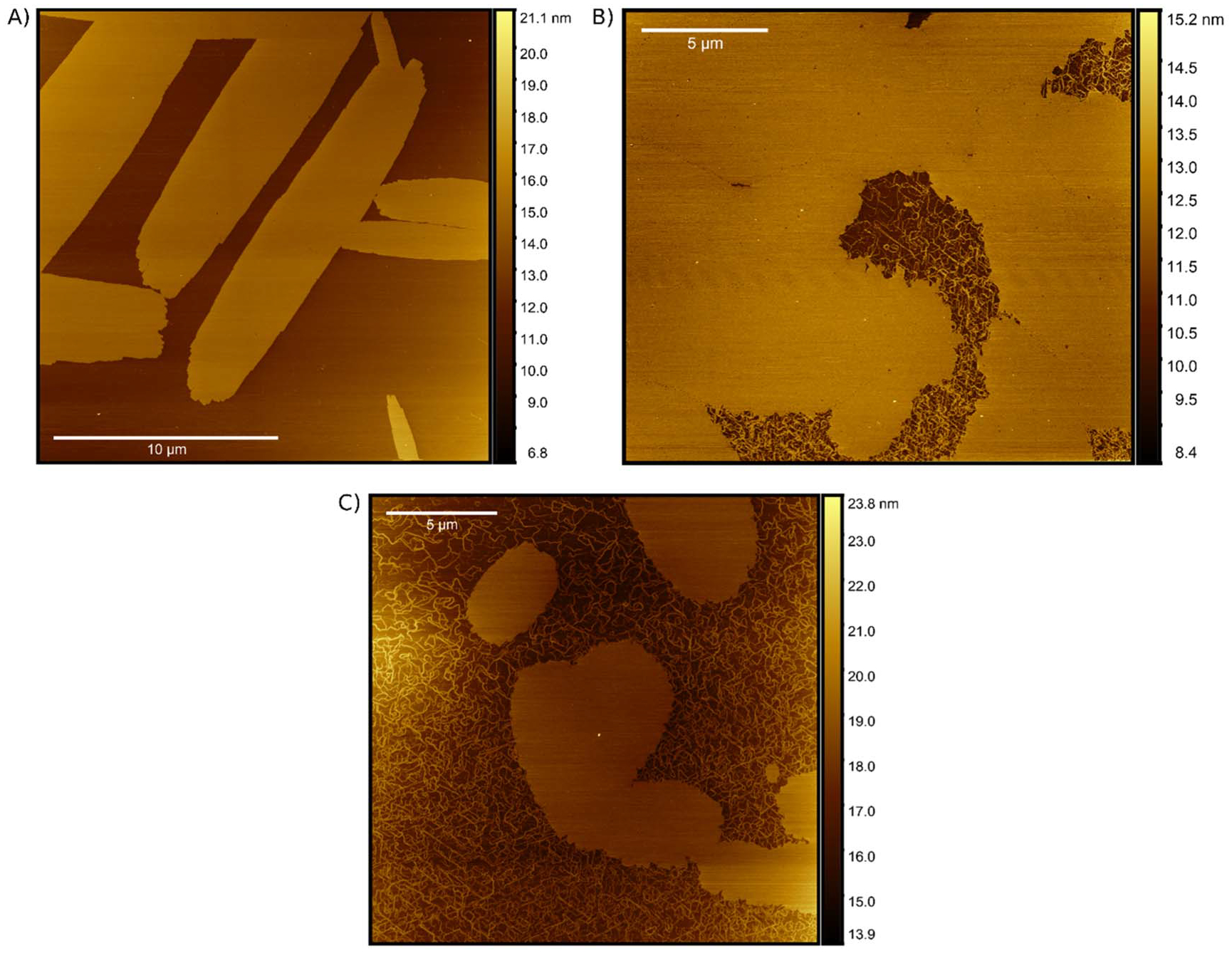
AFM visualization of SA formations. AFM visualizations of PIC aggregates formed in the (**A**) AT, (**B**) dsDNA, and (**C**) ssDNA nanostructures. Each of the samples was formed immediately before measurement by mixing 160 *μ*M PIC dye with 500 nM DNA normalized to the dye-labeled strand concentration (*i.e*. 320-fold excess).

To ensure clean sample preparation, each SA sample was prepared immediately before measurements which lasted approximately 45 min. On this timescale, controls lacking DNA exhibited minimal PIC aggregate formation. Only towards the end of the measurement duration, we observed the appearance of disperse small leafy islands as well as some fibrous PIC chords (Figure S16). Interestingly for all samples except the AT, we were able to observe the growth of leafy islands and aggregates by watching the coalescence of fibrous PIC within the boundaries of the aggregates. When introducing various DNA scaffolds for SA formation and subsequent AFM imaging, we observed significant changes in the aggregates structure. Figure 6 displays example images of PIC SA formed in solution and deposited onto a fresh mica substrate. When the SA was formed using an AT DX-tile template (figures 6(A) and S17) we observed the formation of large and long rod-like aggregates with a relatively isotropic growth axis. This supports the hypothesis proposed by Yoa *et al* which suggested aggregation along preferential axis due to a preferred interaction between the PIC and the mica [70]. Analysis of the SA surface show it had a smooth consistent height limit with a root mean square roughness (*Sq*) of ~122 pm and a surface skewness (*Ssk*) of 0.23. The dimensions of the SA formed between 2 and 4 nm thick, over 20 *μ*m in length, and could be 4 to 5 *μ*m in width. Although the thickness of the SA was in line with the previously measured J-aggregates, the SA had a much larger volume than those formed *sans* DNA [70, 72]. Furthermore, the SA was present immediately upon measurement without fibrous PIC background or leafy islands suggesting these structures were formed in solution prior to deposition on the mica surface.

In contrast to the AT sample the non-DX-tile samples appeared to adopt an entirely different morphology. The dsDNA scaffold (figure 6(B) and figure S18) resulted in a more filmlike PIC formation. In this sample, the film had some similar properties to the SA formed using the AT DX-tile substrate (e.g., *Ssk* = 0.26 and thickness ~2–4 nm) but appeared to lack the directional organization apparent in the AT SA structures. Here, the growth appeared anisotropic allowing the aggregation to propagate outwards relatively consistent in all directions. In addition to the differences in structure, the dsDNA aggregate/film appeared to form less efficiently. The dsDNA aggregate/film had both visible pores and grain boundaries which were not apparent in the AT counterpart. Furthermore, a significant amount of PIC fibers/ribbons were present, appearing with approximately the same thickness as the larger aggregates. These PIC fibers appear to be in line with the structural models proposed by Daltrozzo, *et al* [23] and their 2–3 nm diameter is corroborated by cryogenic transmission electron microscopy (cryo-TEM) measurements by von Berlepsch *et al* [73, 74]. Although this work contributes to the growing body of evidence that solution-based PIC aggregates form fibrous networks structures [73–77], our AFM measurements highlight the multiplicity of PIC aggregation modes when introduced to DNA scaffolds. It appears that in these DNA mixtures, the PIC fibers can further coalesce into larger aggregates using the DNA as nucleation sites. Similar phenomena were also observed in the ssDNA sample (figures 6(C) and S19). However, the ssDNA sample clearly contained a higher proportion of PIC fibers relative to aggregate. Furthermore, the aggregates which were present were smaller in size and lacked the seemingly directed aggregation observed in the AT. These findings appear to correlate nicely with the spectroscopic measurements provided in the preceding sections. The formation of aggregate islands or crystals and the macroscopic structure appears to be correlated with the type of DNA substrate present as well as the observable SA spectroscopic properties.

## Conclusions

In this work we identified a new type of aggregate, the SA, distinct from earlier reported DNA-based PIC aggregates. By utilizing various DNA nanostructure substrates, we were able to elicit divergent features in the resulting SA, which strongly implied differences in the underlying arrangement of the individual PIC molecules. These changes in SA properties appeared to be strongly correlated with both the DNA sequence and the geometry of the fully formed nanostructure. For example, SA formed in rigid DX-tiles appeared to be optimized as light harvesting antennae. Conversely, when formed in more flexible DNA duplex the SA resulted in AF emission quenching, even while maintaining comparable coupling. These differences were then further corroborated with both CD and AFM characterization. The CD spectra of the SAs exhibited distinct spectral signatures relative to both previously characterized PIC J-bits [22, 30–33, 50] as well as between the DNA substrates. Finally, the AFM imaging highlighted the stark differences in aggregate formation resulting from the DNA substrates. To our knowledge this is the first visualization of DNA-based PIC aggregates. Results from the field have been pointing towards fiber-like or nanotube-like networks of polymerized PIC as a structural model for aggregates suspended in solution [8, 11, 15, 23, 50, 51, 73–76]. On the other hand, other AFM studies demonstrate that PIC aggregates formed on mica substrates adopt a leafy island morphology [70–72]. Interestingly, we observe evidence of both PIC fibers as well as leafy islands that exhibit distinct growth patterns, again depending on the DNA substrate. Considering each of the results collectively, these observations add to the existing body of work in that we show the space of interesting DNA nucleated PIC aggregates are not limited to J-bit simply templated in AT-tracks. Bearing in mind that the broader motivation for studying DNA based PIC aggregates is to integrate strongly coupled dyes onto modular DNA structural units, PIC SAs should be given due consideration as a versatile option. In fact, similar work is being done with other cyanine dyes where DNA template modification is used to switch between quenching and energy transfer [10, 34, 55]. Ultimately this could be a path for the PIC SA and one which possibly leads towards applications in optical microcavities for quantum electrodynamical devices and optical switching, [42, 78–80] molecular plasmonics, [81–84] biosensors, [85] and light-harvesting arrays [49, 86, 87].

## Supplementary Material

Supplementary Material

## Figures and Tables

**Table 1.** Relative change (*R*_c_) in PIC emission and estimated PIC fluorescence QY (Φ) utilizing 523 nm excitation and in AF emission and QY using a 647 nm excitation.

Sample	PIC (523 nm excitation)		AF (647 nm excitation)	
	*R*_*c*_ (%)	Φ (%)	*R*_*c*_ (%)	Φ (%)
AT	15.4 ± 1.5	0.23 ± 0.03	−80.8 ± 2.6	12.8 ± 4.6
GC	−25.7 ± 0.6	0.15 ± 0.02	−84.6 ± 1.4	12.1 ± 2.5
dsDNA	−40.8 ± 1.1	0.12 ± 0.02	−95.5 ± 0.2	4.8 ± 0.9
ssDNA	−43.7 ± 0.6	0.10 ± 0.01	−95.9 ± 0.3	6.1 ± 3.6
PIC (monomer)	−74.3 ± 0.2	0.04 ± 0.01	—	—
J-bit (Reference)	—	0.18 ± 0.03	—	—
Free AF	—	—	−2.6 ± 0.7	33.0 ± 3.3

* DNA sequences and structures are available in figures S1–S4.

**Table 2.** Relative change in AF emission and estimated energy transfer from PIC to AF utilizing 580 nm excitation.

Sample	*R*_*c*_ (%)	ET
AT	195 ± 2	0.65 ± 0.17
GC	131 ± 2	0.21 ± 0.05
dsDNA	−33.3 ± 0.2	0.24 ± 0.06
ssDNA	−81.4 ± 0.1	0.08 ± 0.06

* DNA sequences and structures are available as in figures S1–S4.

## Data Availability

All data that support the findings of this study are included within the article (and any supplementary files).
