# Supplementary material for: Structural and optical variation of pseudoisocyanine aggregates nucleated on DNA substrates
Source: Methods Appl Fluoresc. Author manuscript; Available in PMC 2023 Jul 22. (PMC10362908; doi:10.1088/2050-6120/acb2b4)
Supplement: Supplementary Material [file NIHMS1915820-supplement-Supplementary_Material.pdf]

Structural and Optical Variation of Pseudoisocyanine Aggregates Nucleated on DNA Substrates

Matthew Chiriboga [1,2], Christopher M. Green [1], Divita Mathur [1,3], David Hastman [1], Joseph Melinger [4], Remi Veneziano [2], Igor L. Medintz [1], Sebastián A Díaz [1*]

[1]Center for Bio/Molecular Science and Engineering Code 6900
U.S. Naval Research Laboratory
4555 Overlook Ave. S.W.
Washington, DC 20375, USA

[2] Department of Bioengineering
Volgenau School of Engineering
George Mason University
4400 University Drive
Fairfax, VA 22030, USA

[3]Department of Chemistry
Case Western Reserve University
10900 Euclid Avenue
Cleveland, OH 44106, USA

[4]Electronics Sciences and Technology
U.S. Naval Research Laboratory
4555 Overlook Ave. S.W.
Washington, DC 20375, USA

*Corresponding Author
Email: Sebastian.Diaz@nrl.navy.mil

Contents:

Supplemental Methods	Page
**Spectra Processing and Correction Methods**	
Free AF Spectra Correction	S-3
J-Bit Spectra Correction	S-3
Inner Filter Correction	S-4
Curve Smoothing	S-4
Error Propagation	S-5
Quantum Yield Estimation	S-5
Spectral Overlap Calculation	S-6
Förster Distance Calculation	S-6
**DNA Nanostructure Data**	
Figure S1: AT Substrate	S-8
Figure S2: GC Substrate	S-9
Figure S3: dsDNA Substrate	S-10
Figure S4: ssDNA Substrate	S-11
**Supplemental Spectra**	
Figure S5: PIC Aggregate Excitation Spectra	S-12
Figure S6: Normalized Component Absorption/Emission Spectra	S-13
Figure S7: PIC (Monomer) vs DNA+ PIC Full Absorbance Spectra	S-14
Figure S8: Negative Control Full Absorbance Spectra	S-14
Figure S9: PIC vs Free AF Absorbance Spectra	S-15
Figure S10: DNA+AF vs Free AF Absorbance Spectra	S-15
Figure S11: SA vs PIC (Monomer) Absorbance Spectra	S-16
Figure S12: SA vs Free AF Absorbance Spectra	S-17
Figure S13: SA vs Free AF Absorbance Spectra Normalized	S-18
Figure S14: 647 nm Excitation Inner Filter	S-19
Figure S15: CD and Absorbance Temperature Dependence	S-20
**Supplemental AFM images and analysis**	
Figure S16: PIC Only AFM	S-21
Figure S17: AT+PIC AFM Analysis	S-22
Figure S18: dsDNA+PIC AFM Analysis	S-23
Figure S19: ssDNA+PIC AFM Analysis	S-24
**References**	

Spectra Processing:

Free AF Concentration Correction.

Raw absorbance spectra were collected in triplicate as outlined in the *Methods* section of the main text and averaged to make the presented experimental spectra. After averaging, each spectrum was adjusted for baseline shifts by subtracting the minimum absorbance from the spectrum elementwise. Next, each spectrum was then corrected for background by subtracting the buffer only control from each spectrum. Due to incomplete mixing, Free AF647 samples were measured to be at a lower concentration. To correct for this difference in concentration, the DNA+AF controls i.e., AT+AF, GC+AF, dsDNA+AF, and ssDNA+AF were averaged to provide a reference AF spectrum. The correction factor α was then calculated by

$$\alpha = \frac{A_{max}^{ref}}{A_{max}^{Free\ AF}} \qquad \text{Eq. S1}$$

where A_{max}^{ref} and $A_{max}^{Free\ AF}$ refer to the maximum absorbance of the reference spectra and the Free AF spectra respectively. The Free AF absorbance spectrum was then scaled by α which was approximately 1.24. Similarly, the Free AF+PIC spectrum was also scaled by α when visualizing the AF peak.

J-Bit Concentration Correction.

Similarly, raw fluorescence emission spectra were collected in triplicate as outlined in the *Methods* section of the main text and averaged to make the presented experimental spectra. Firstly, each emission spectrum was corrected for baseline shifts. Next, the Free AF and Free AF+PIC spectra were scaled by α to account for the difference in concentration. Finally, the J-bit spectrum was corrected for the difference in PIC concentration required to form the J-bit compared to the SA. The correction factor γ was calculated by taking the ratio of the concentrations. The J-bit is formed at 120-fold excess of PIC to dye labeled DNA while the SA is formed at a 320-fold excess. In each case, the dye labeled DNA strand was constant at 500 nM, meaning the PIC dye

concentration was 60 µM and 160 µM for J-bit and SA samples respectively. Therefore, γ was calculated as

$$\gamma = \frac{160\ \mu\text{M}}{60\ \mu\text{M}} \qquad \text{Eq. S2}$$

and the J-bit+PIC sample was then scaled by γ.

Inner Filter Correction.

Since highly concentrated samples of PIC dye are necessary to from SA, we included a correction for inner filter effects (β). This correction is in order to account for the non-negligible attenuation of the excitation beam through the path of the sample. The inner filter component can be isolated by comparing the Free AF and Free AF+PIC controls and taking the ratio of their integrated emission. β can be calculated for arbitrary excitation wavelength according to

$$\beta = \frac{\Psi^{AF}}{\Psi^{AF+PIC}} \qquad \text{Eq. S3}$$

where Ψ is integrated emission and the superscripts AF and AF+PIC refer to the Free AF and Free AF+PIC samples. The samples with PIC dye present are then scaled by β. For 647 nm excitation there was minimal apparent inner filter where β was approximately 1.02. Logically this makes sense as PIC dye absorbs minimally above 615 nm. However, PIC has a much higher absorption cross section at 580 nm resulting in a larger required β factor of approximately 1.35.

Curve Smoothing.

Spectra in the manuscript figures were smoothed for presentation using a simple 1-dimensional moving average described by

$$p_\lambda^s = \frac{1}{k}\sum_{i=\lambda}^{\lambda+k} p_i \qquad \text{Eq. S4}$$

where p_λ^s is the smoothed value at wavelength λ, k is the window size or sampling size, and p_i is the pre-smoothed value. This operation is then repeated for each wavelength measured in the spectra.

Edge positions (i.e. the last 2 wavelengths) are simply used as is. The spectra in main text figures were smoothed using a window size of three.

Error Propagation.

Each sample was measured with $n = 3$ repeats and the sample error (σ) was calculated by taking the sample standard deviation according to

$$\sigma = \frac{1}{n-1}\sum_{i=1}^{n}(x_i - \bar{x})^2 \qquad \text{Eq. S5}$$

where x_i is the measured value of repeat i, and $\bar{x}$ is the mean of the three repeats. The error was propogated through arithmatic steps in quadrature. For addition and subtraction of measurements (*x*, *y*) with associated error (*σx*, *σy*) , the propogated error (*σz*) was calculated according to

$$\sigma z = \sqrt{\sigma x^2 + \sigma y^2} \qquad \text{Eq. S6}$$

while for multiplication and division steps, the relative error was propogated according to

$$\sigma z = z \times \sqrt{\frac{\sigma x^2}{x} + \frac{\sigma y^2}{y}} \qquad \text{Eq. S7}$$

where *z* is the product or quotient of *x*, *y*.

Quantum Yield Estimation.

Quantum yield (QY) estimations were calculated for both PIC and AF. In this case the quantum yields of both dyes were estimated relative to themselves, in conditions which were previously measured relative to a primary QY standard. The QY of AF647 attached to the AT DX-tile in tris buffer was previously measured to be 41 ± 2.6% relative to oxazine 710 in methanol [1]. The QY of J-bits templated in 8-mer AT-track DX-tile in tris buffer was calculated to be 0.18 ± 0.03% relative to Rhodamine 6G [2]. Using these references, the QY estimate of the sample (Φ_s) was calculated according to

$$\Phi_s = \Phi_{ref} \times \frac{\Psi_s}{\Psi_{ref}} \times \frac{1-10^{-A_{ref}}}{1-10^{-A_s}} \times \frac{n_s^2}{n_{ref}^2} \qquad \text{Eq. S8}$$

where Φ denotes QY, Ψ denotes integrated emission, A denotes absorbance at the excitation wavelength, n denotes the refractive index of the measurement medium, and the subscripts s and ref denote the sample in question and the reference respectively. Since both the sample and reference were both measured in identical buffer, the final refractive index correction is therefore 1. The excitation wavelengths of 523 nm and 647 nm were used to calculate the QY for PIC and AF647 respectively.

Spectral Overlap Calculations.

Spectral overlap between the PIC dye (monomeric, J-bit and SA) and AF647 were calculated using experimental spectra. Each J-integrand (J_λ) was calculated according to

$$J_\lambda = \int_{\lambda_i}^{\lambda_f} F_D(\lambda)\varepsilon_A(\lambda)\lambda^4 d\lambda \quad \text{Eq. S9}$$

where λ is a given wavelength, $F_D(\lambda)$ is the normalized donor fluorescence measured at wavelength λ such that the total donor fluorescence is unity, $\varepsilon_A(\lambda)$ is the extinction of the acceptor at wavelength λ using the manufacturer reported max extinction of AF647 as 270,000 $cm^{-1}M^{-1}$, and the integral is taken from the starting wavelength λ_i to λ_f.

Förster Distance Calculation.

The Förster distances (R_0) between PIC (monomer, J-bit, and SA) were calculated according to FRET theory using experimentally obtained spectra and quantum yield estimates. To

arrive at a logical interpretation of Förster distance, we consider PIC aggregate to be a molecular unit, signifying that we assume the point-dipole approximation is still valid. The Förster distance was calculated in nm according to

$$R_0 = (\frac{9\ln(10)}{128\pi^5 N_A} \times \frac{\kappa^2 \Phi_D}{n^4} \times 10^{17} \times J_\lambda)^{\frac{1}{6}} \quad \text{Eq. S10}$$

where N_A is Avogadro's number ($N_A = 6.02 \times 10^{23}$), κ^2 is the dipole orientation factor assumed to be $\frac{2}{3}$, Φ_D is the quantum yield of the donor and is assumed to be 0.00012 for monomeric PIC [3], 0.0018 for J-bit [2], and 0.0023 for SA formed with the AT substrate, n is the refractive index of the measurement medium and $n = 1.33$ for aqueous measurements, and J_λ is the overlap integral calculated with respect to each donor species as outlined above.

DNA Nanostructure Data.

AT:

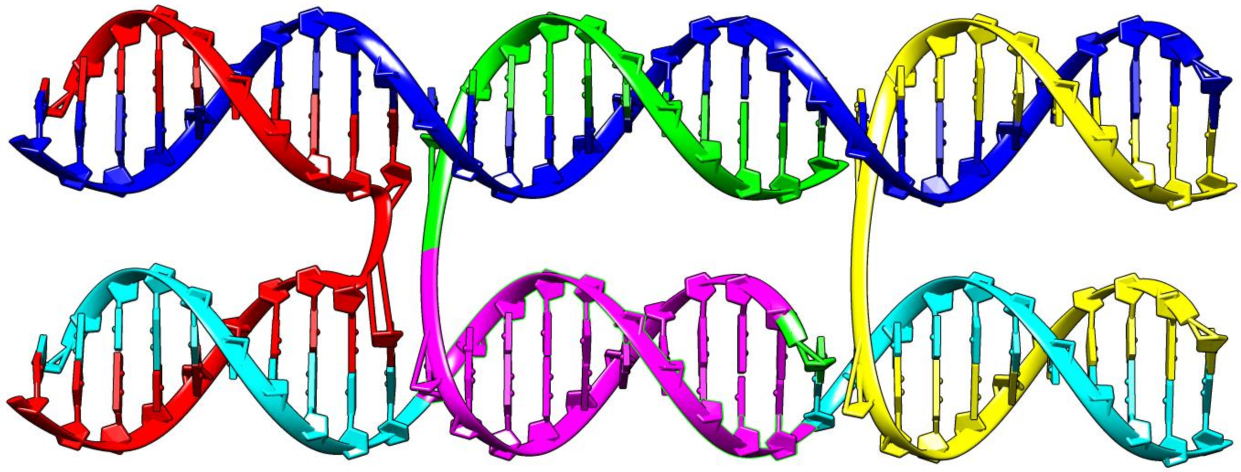

AT	sequence	length	gc (%)	mw (g/mol)	ε_{260} (M^{-1} cm^{-1})	T_m (C)
Strand 1	TCAGAAGAACAAAAAAAAAA	20	20	6177.17	228200	59
Strand 2	ACGCATTTGTCGTTCTTCTGATGACCTTGCA	31	45	9443.19	282000	76
Strand 3	GTACACCTCGCTTTTTTTTTTGTGTCTGCAA	31	39	9424.19	275100	74
Strand 4	TTGCAGACACGACAAATGCGT	21	48	6439.26	206500	70
Strand 5	TGCAAGGTCCGCGAGGTGTAC	21	62	6487.26	202300	75

Figure S1: Structure and sequence data for the AT DX-tile substrate. The AT DX-tile nanostructure, self-assembled from 5 ssDNA oligonucleotides. The strand highlight color-coding is to match the sequence to the strand location in the nanostructure. The magenta sections of Strand 1 and Strand 3 denote the non-alternating poly(AT) sequence (AT-track). The strand sequence is accompanied by various properties predicted from the sequence information and our experimental conditions using an open source oligo-analyzer (https://github.com/mchirib1/DNA-Oligo-Analysis).

GC:

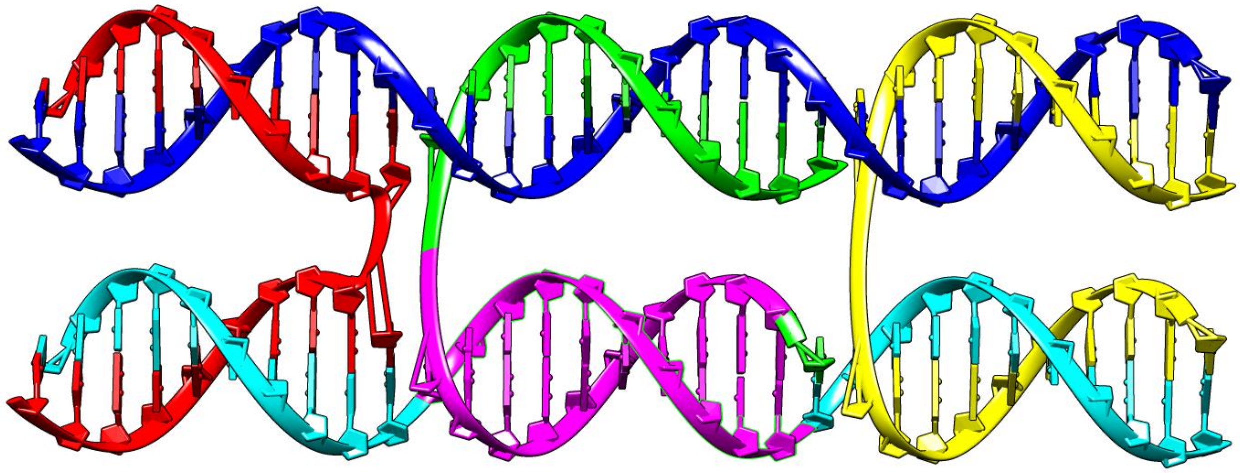

GC	sequence	length	gc (%)	mw (g/mol)	ε_{260} (M^{-1} cm^{-1})	T_m (C)
Strand 6	TCAGAAGAACGCGCGCGCGC	20	70	6137.02	189900	76
Strand 2	ACGCATTTGTCGTTCTTCTGATGACCTTGCA	31	45	9443.19	282000	76
Strand 7	GTACACCTCGAGCGCGCGCGCTTGTCTGCAA	31	65	9473.16	281600	82
Strand 8	TTGCAGACAAGACAAATGCGT	21	43	6463.29	211700	69
Strand 5	TGCAAGGTCCGCGAGGTGTAC	21	62	6487.26	202300	75

Figure S2: Structure and sequence data for the GC DX-tile substrate. The AT DX-tile nanostructure, self-assembled from 5 ssDNA oligonucleotides. The strand highlight color-coding is to match the sequence to the strand location in the nanostructure. The magenta sections of Strand 1 and Strand 3 denote the alternating poly(GC) sequence (GC-track). The strand sequence is accompanied by various properties predicted from the sequence information and our experimental conditions using an open source oligo-analyzer (https://github.com/mchirib1/DNA-Oligo-Analysis).

dsDNA:

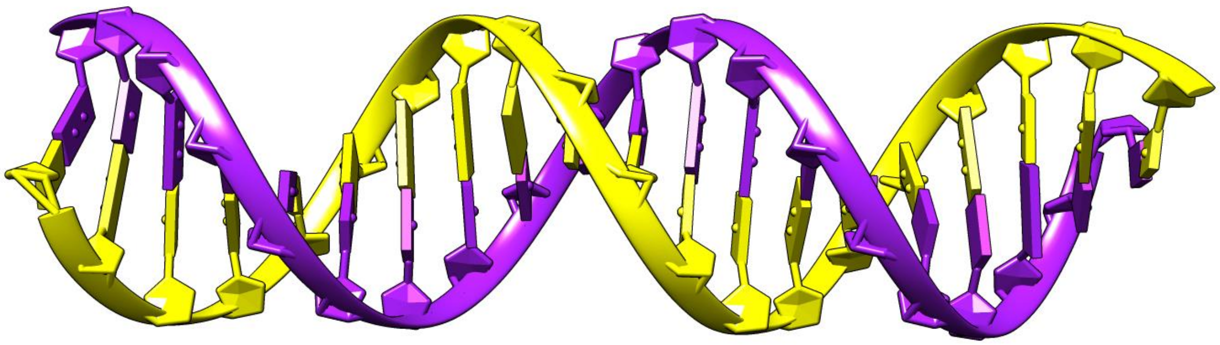

dsDNA	sequence	length	gc (%)	mw (g/mol)	ε_{260} (M^{-1} cm^{-1})	T_m (C)
Strand 5	TGCAAGGTCCGCGAGGTGTAC	21	62	6487.26	202300	75
Strand 9	ACGTTCCAGGCGCTCCACATG	21	62	6367.17	193000	75

Figure S3: Structure and sequence data for the dsDNA substrate. The dsDNA nanostructure, assembled from two ssDNA oligonucleotides. The strand highlight color-coding is to match the sequence to the strand location in the nanostructure. The strand sequence is accompanied by various properties predicted from the sequence information and our experimental conditions using an open source oligo-analyzer (https://github.com/mchirib1/DNA-Oligo-Analysis).

AF647 labeled ssDNA:

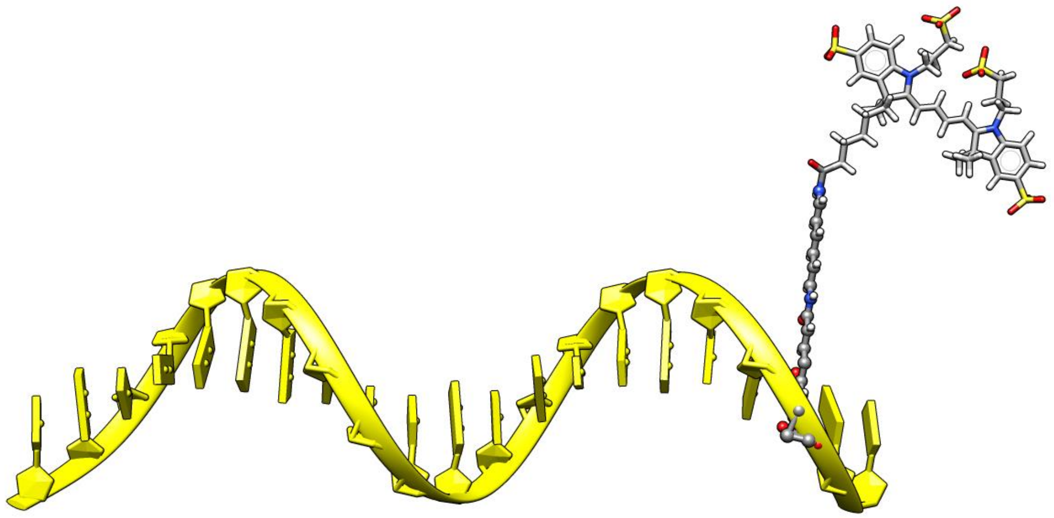

ssDNA	sequence	length	gc (%)	mw (g/mol)	ε_{260} (M^{-1} cm^{-1})	T_m (C)
Strand 5-AF	TGCAAGGTCCGCGAGGTG\T-*C6:AF647* \AC	21	62	6487.26	202300	75

Figure S4 Structure and sequence data for the ssDNA substrate. The ssDNA nanostructure consists of only a single strand in solution. The strand sequence is accompanied by various properties predicted from the sequence information and our experimental conditions using an open source oligo-analyzer (https://github.com/mchirib1/DNA-Oligo-Analysis). The figure and sequence also reflect the AF647 modification, which would be present for Strand 5 in structures which included the AF reporter dye. In order to attach the dye an internal Amino Modifier C6 dT is integrated into the sequence. Then AlexaFluor 647 Maleimide is attached to the amino terminated C6 linker through NHS ester chemistry.

Supplemental Spectra:

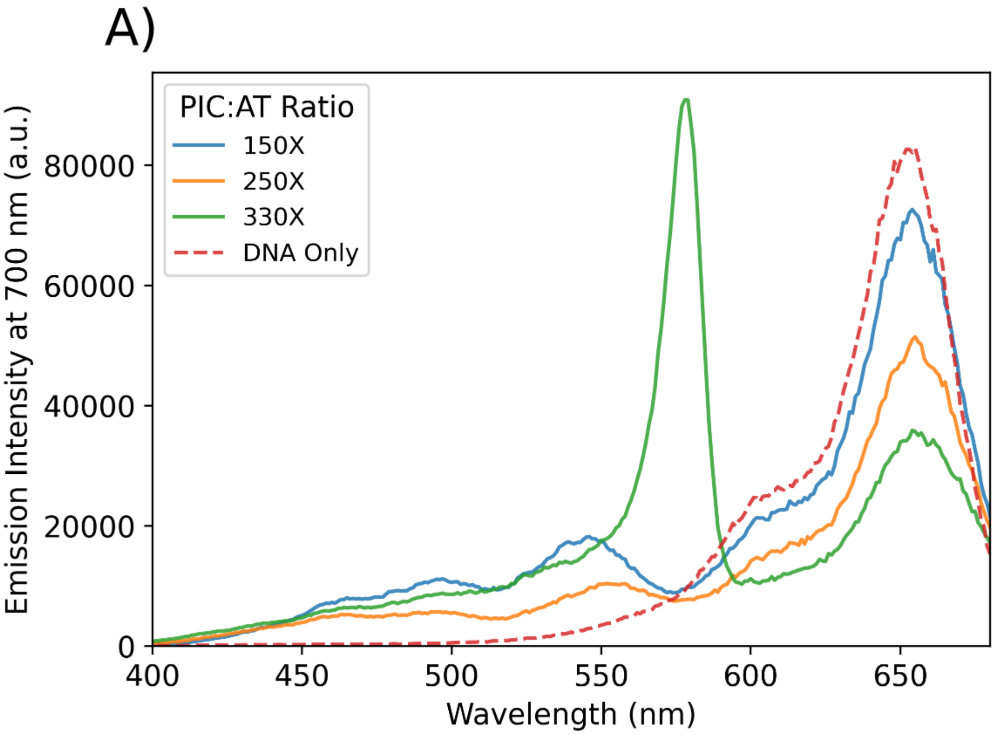

Figure S5: Excitation spectra of AT+PIC+AF at different PIC:DNA ratios versus DNA only control. Excitation spectra of the AT+PIC+AF structure with 150-fold (blue), 250-fold (orange), and 330-fold (green) excess PIC to DNA. The AT+AF negative control was included as a reference. The emission at 700 nm is measured as a function of excitation wavelength. In each sample DNA concentration is at 400 nM. It is interesting to note that the AF concentration is constant in all samples and as discussed on Page S-4 the internal filter effect is negligable at excitation wavelengths above 615 nm. As such the decrease of the peak at 650 nm must be due to a decrease in the AF QY though coupling to PIC aggregates as discussed in the main text.

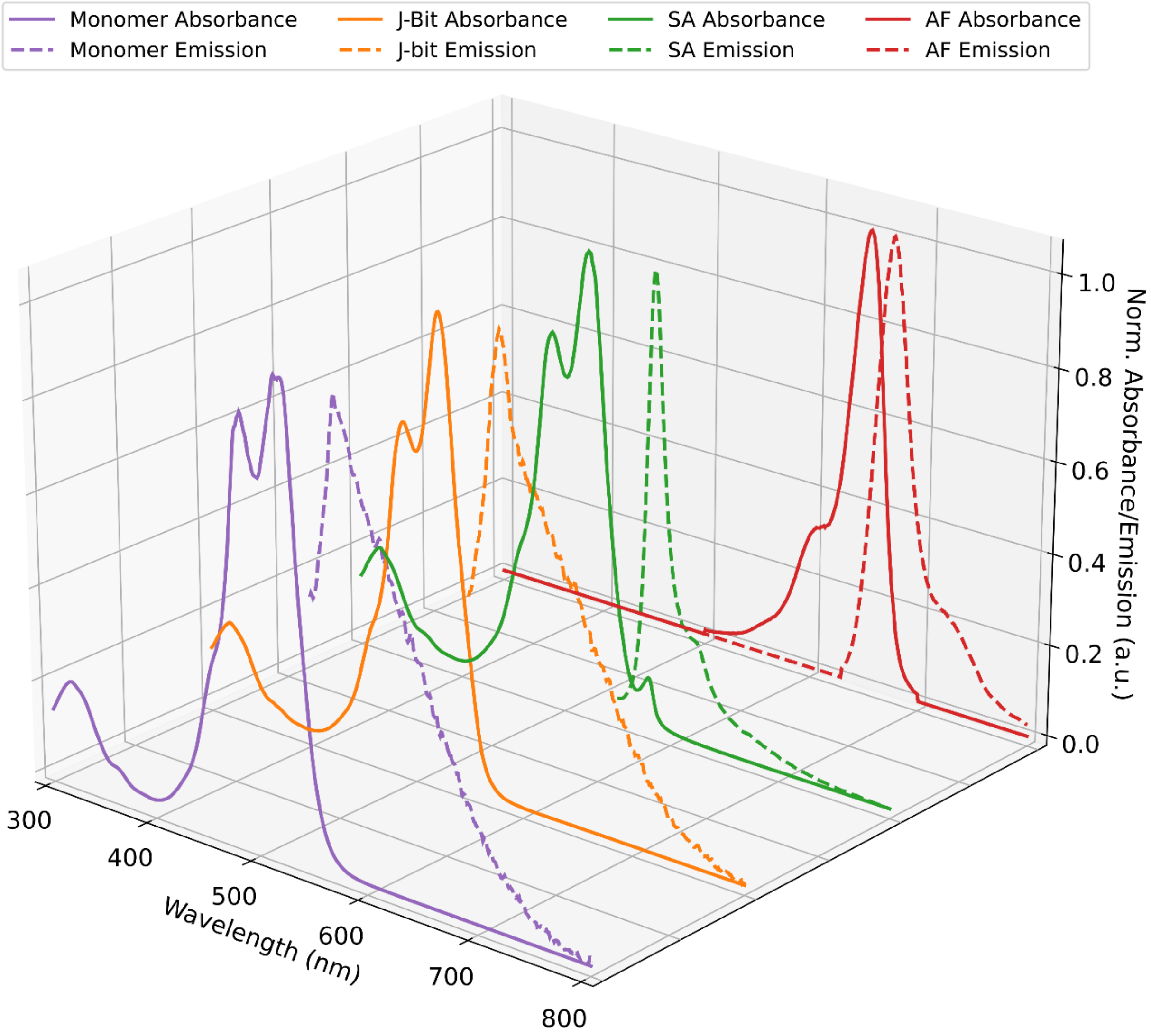

Figure S6: Fluorophore photophysical properties. Normalized absorption and emission spectra for PIC monomer, J-bit, SA (AT+PIC), and AF.

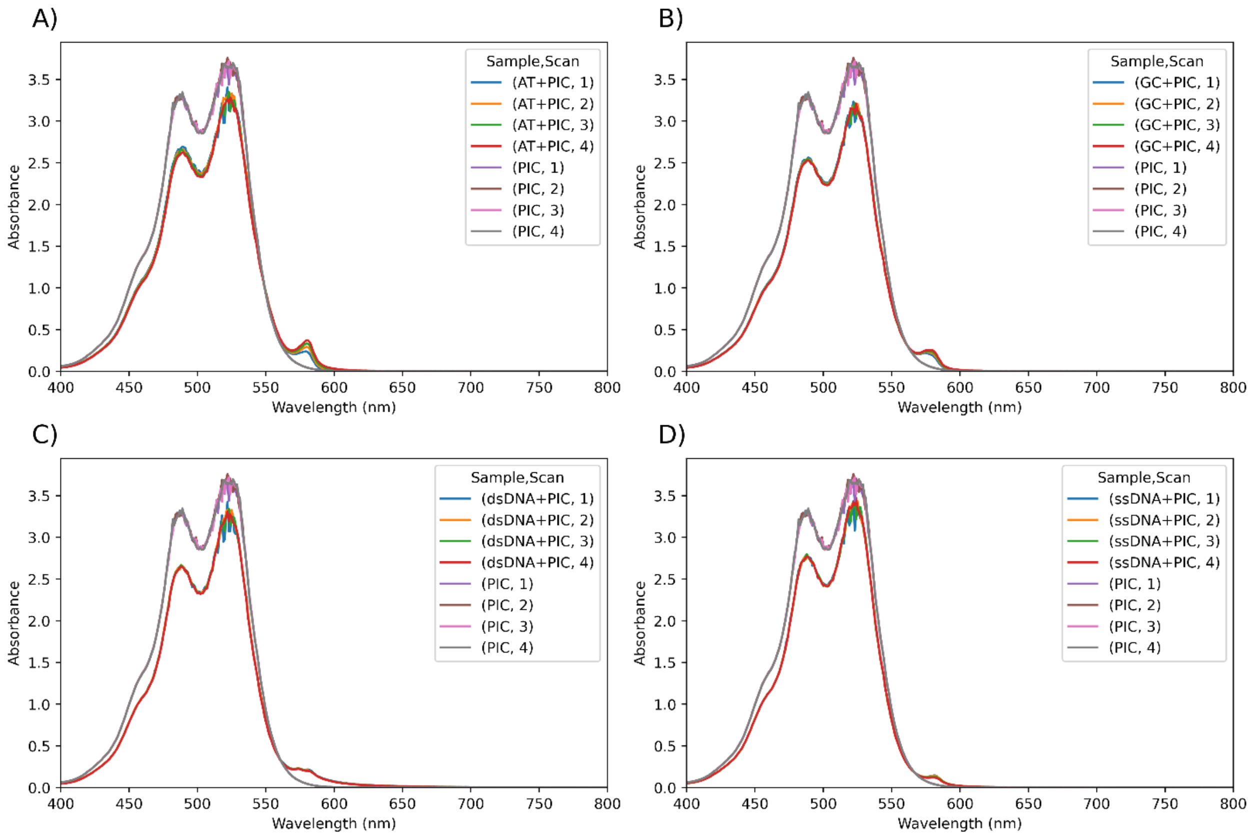

Figure S7: Full absorbance spectra of DNA+PIC versus PIC (monomer). (A) AT+PIC, **(B)** GC+PIC, **(C)** dsDNA+PIC, and **(D)** ssDNA+PIC, versus PIC only control which is considered to be monomeric. The scan number refers to the individual measurement time point with one being measurement immediately after sample preparation and four is approximately 36 hours after preperation. In each sample DNA concentration is at 500 nM, while the PIC dye is in 320-fold excess (160 µM). The SA formation in the absence of AF labeled DNA confirms the SA is not a PIC-AF specific interacion.

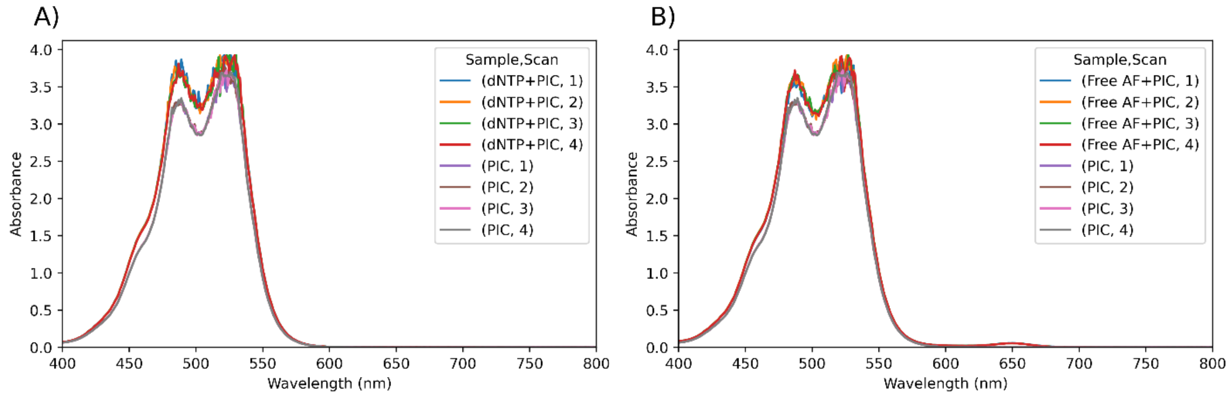

Figure S8: Full absorbance spectra of PIC (monomer) versus negative controls dNTP+ PIC and Free AF+PIC. No SA peak is observed in the spectra. The scan number refers to the individual measurement time point with one being measurement immediately after sample preparation and four is approximately 36 hours after preperation. The (A) Free AF and (B) dNTPs are at final concentrations of 500 nm.

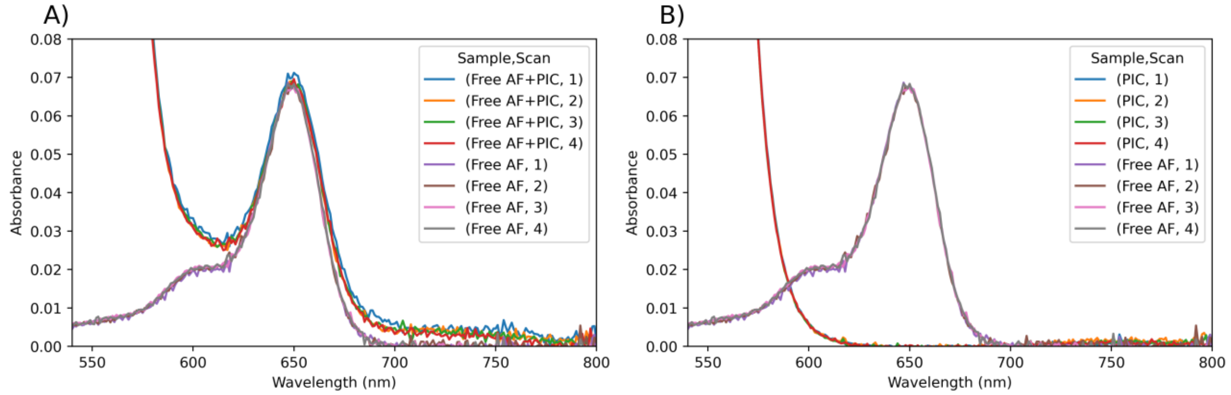

Figure S9: Absorbance spectra focused on the AF647 peak of Free AF versus Free AF+PIC and PIC (Monomer). The scan number refers to the individual measurement time point with one being measurement immediately after sample preparation and four is approximately 36 hours after preperation. In each sample the Free AF concentration are at 500 nM and the PIC dye is in 320-fold excess (160 μM). The Free AF spectra were corrected by a factor of α to in order to account for incomplete solvation of the dye powder targeted to be 500 nM.

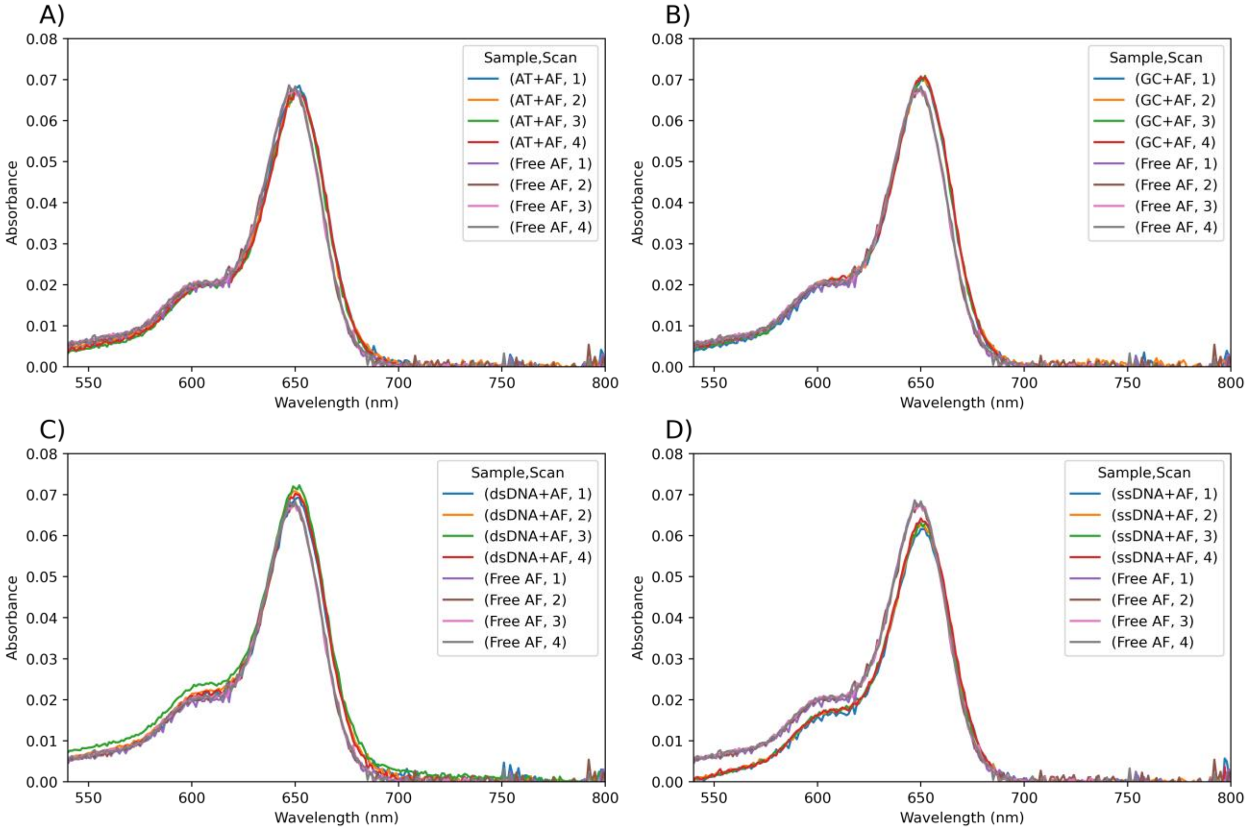

Figure S10: Absorbance spectra focused on the AF647 peak of DNA+AF versus Free AF. (A) AT+AF, (B) GC+AF, (C) dsDNA+AF, and (D) ssDNA+AF, versus Free AF. The scan number refers to the

individual measurement time point with one being measurement immediately after sample preparation and four is approximately 36 hours after preperation. In each sample DNA concentration is at 500 nM. The Free AF spectra were corrected by a factor of α to in order to account for incomplete solvation of the dye powder targeted to be 500 nM. Integration of the AF into the DNA structures did not modify its properties.

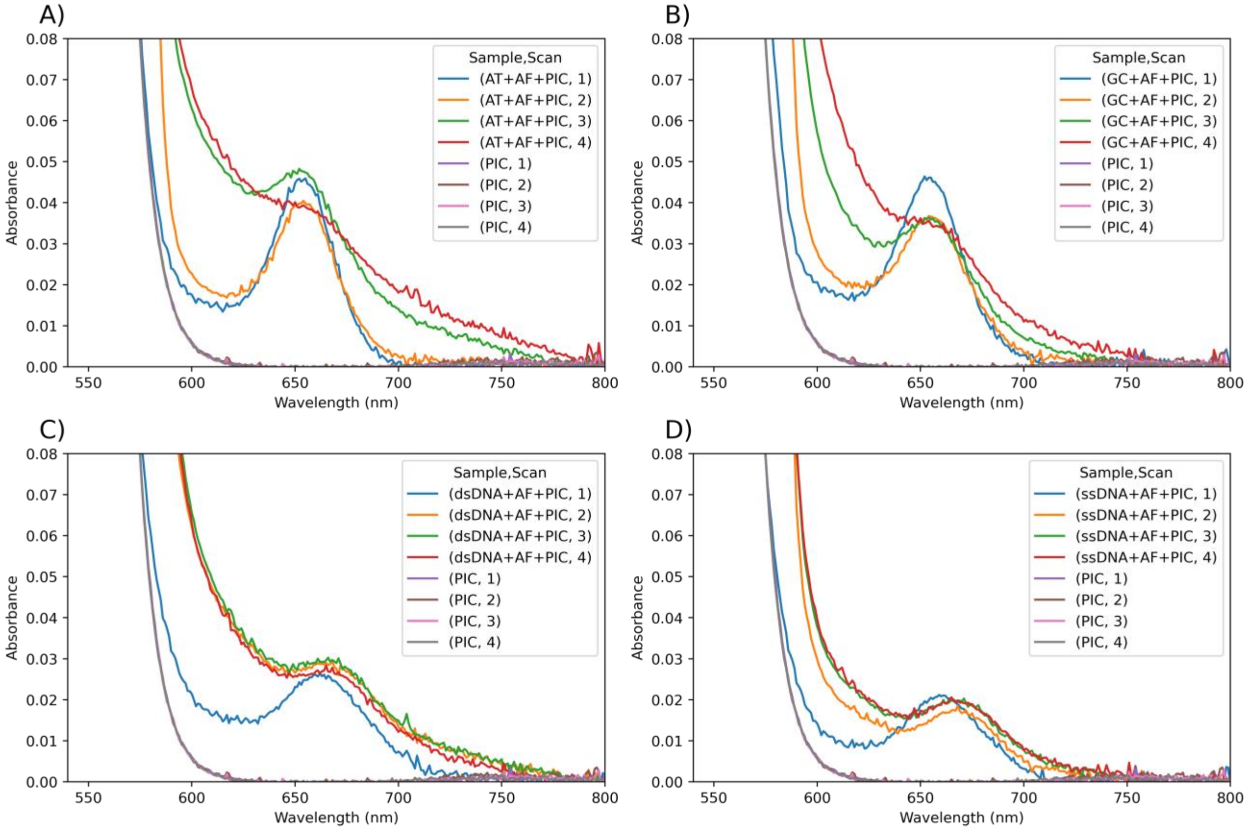

Figure S11: Absorbance spectra focused on the AF647 peak of DNA+AF+PIC versus PIC (Monomer). (A) AT+AF+PIC, (B) GC+AF+PIC, (C) dsDNA+AF+PIC, and (D) ssDNA+AF+PIC, versus PIC only control which is considered to be monomeric. The scan number refers to the individual measurement time point with one being measurement immediately after sample preparation and four is approximately 36 hours after preperation. In each sample DNA concentration is at 500 nM, while the PIC dye is in 320-fold excess (160 μM).

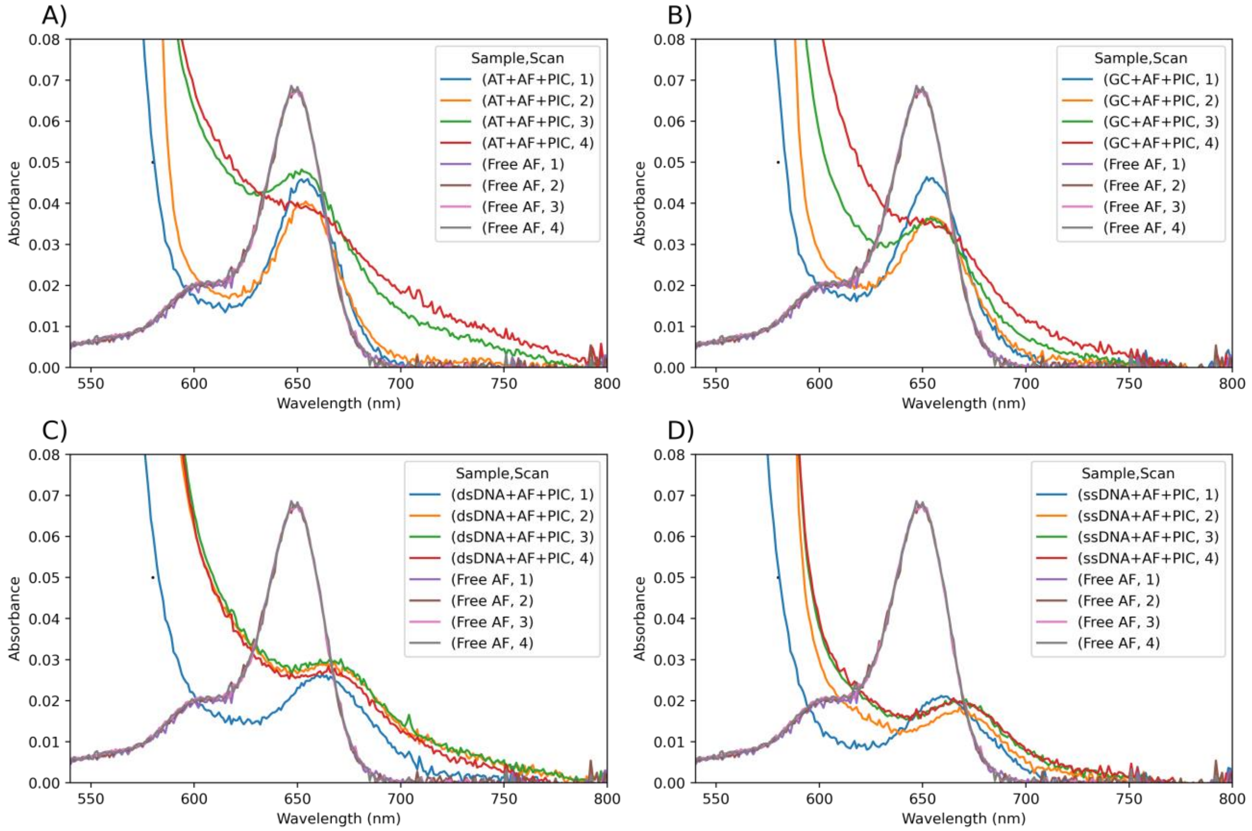

Figure S12: Absorbance spectra focused on the AF647 peak of DNA+AF+PIC versus Free AF. (A) AT+AF+PIC, (B) GC+AF+PIC, (C) dsDNA+AF+PIC, and (D) ssDNA+AF+PIC, versus Free AF. The scan number refers to the individual measurement time point with one being measurement immediately after sample preparation and four is approximately 36 hours after preperation. In each sample DNA concentration is at 500 nM, while the PIC dye is in 320-fold excess (160 μM). The Free AF spectra were corrected by a factor of α to in order to account for incomplete solvation of the dye powder targeted to be 500 nM.

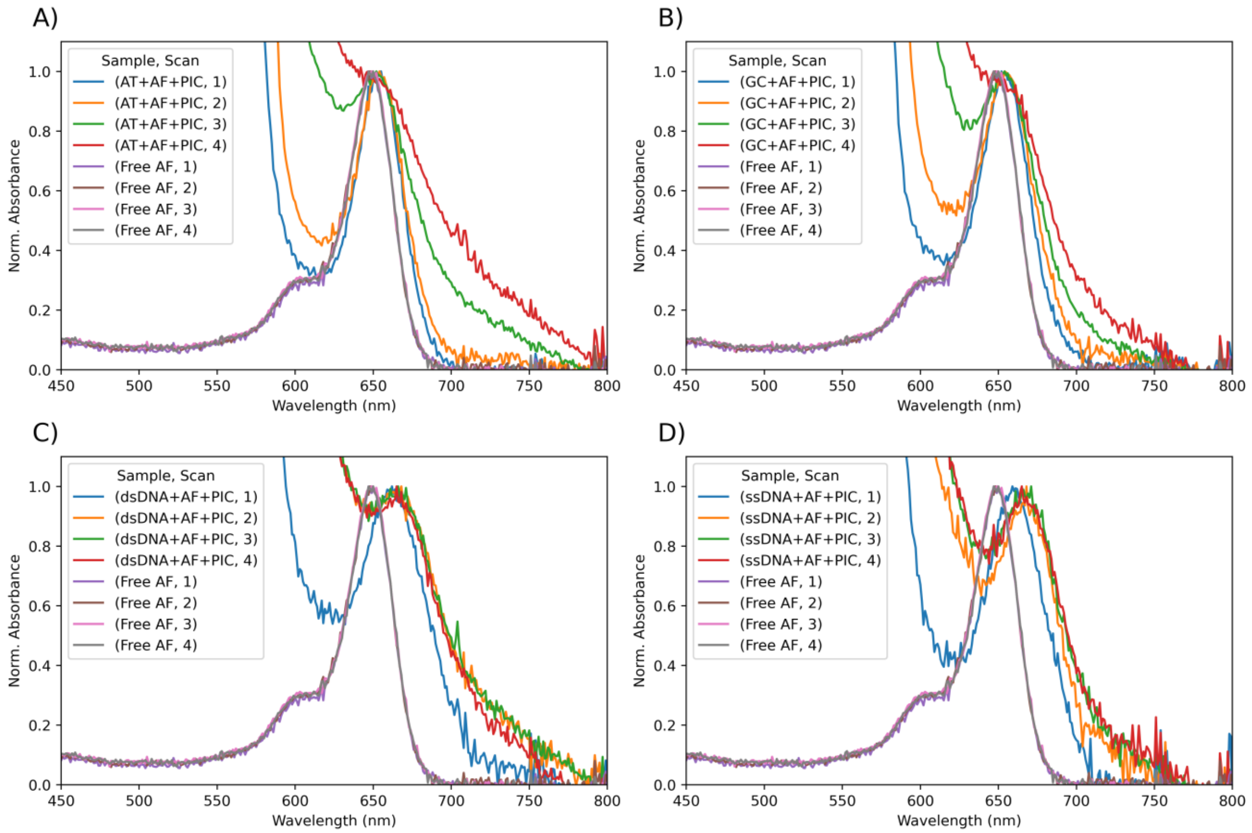

Figure S13: Normalized absorbance spectra from Figure S12. The intent is to show the peak broadening and shift. (A) AT+AF, (B) GC+AF, (C) dsDNA+AF, and (D) ssDNA+AF, versus Free AF. Each spectrum is normalized such that the AF647 peak is unity. The scan number refers to the individual measurement time point with one being measurement immediately after sample preparation and four is approximately 36 hours after preperation. In each sample DNA dye labeled strand and the Free AF concentration are at 500 nM.

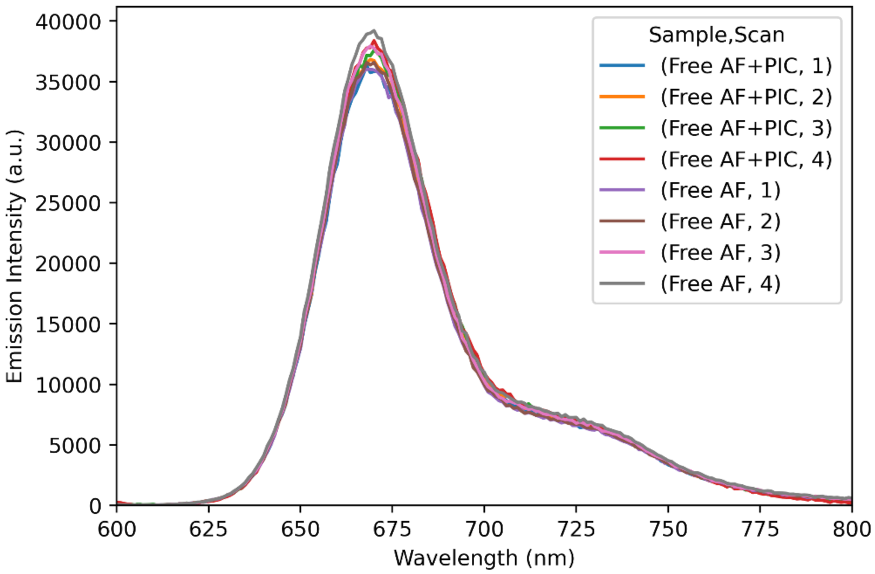

Figure S14: Fluorescence emission spectra using a 647 nm excitation wavelength of Free AF+PIC versus Free AF. The scan number refers to the individual measurement time point with one being measurement immediately after sample preparation and four is approximately 36 hours after preperation. The Free AF spectra were corrected by a factor of α to in order to account for incomplete solvation of the dye powder targeted to be 500 nM.

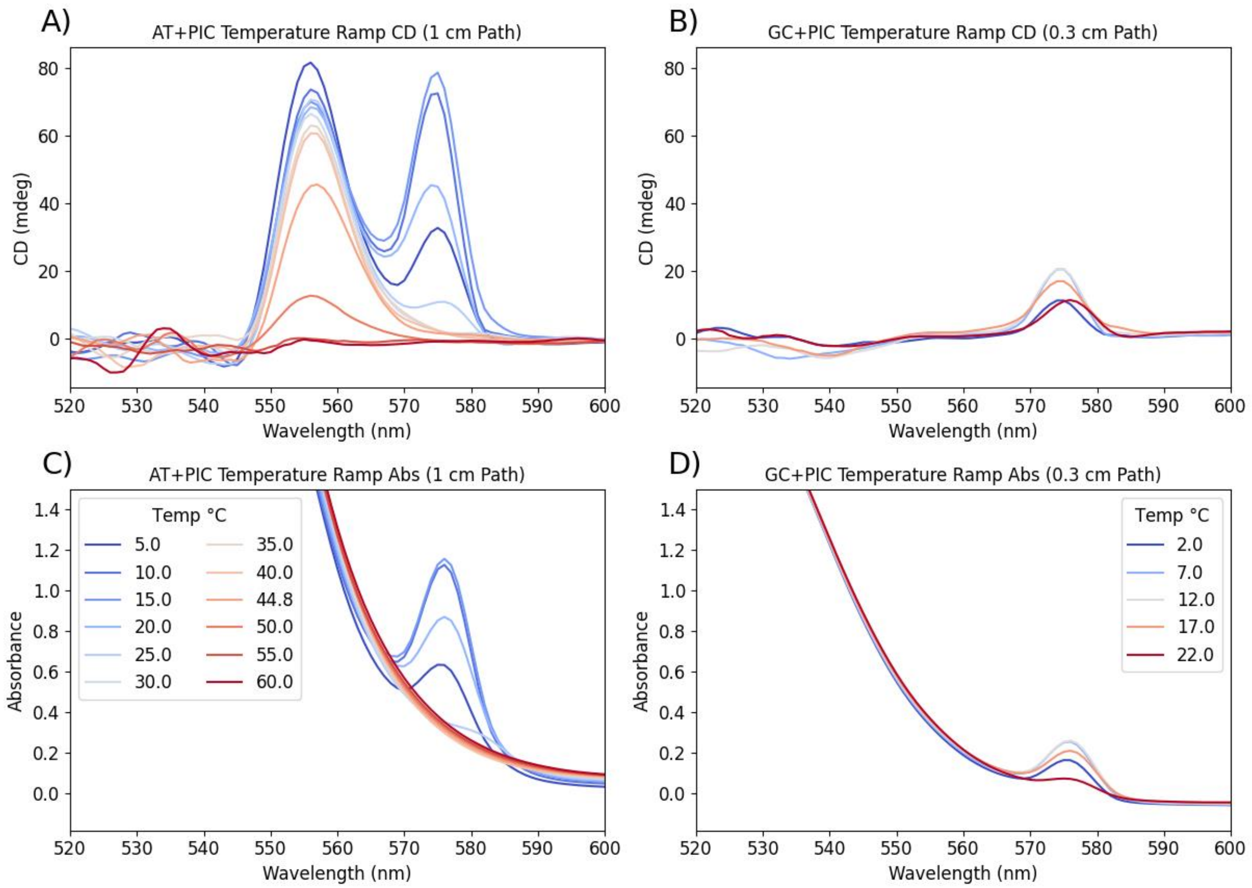

Figure S15: Temperature dependant circular dichroism and absorbance spectra of AT+PIC and GC+PIC. The AT+PIC sample was prepared at room temperature and allowed to equilibrate to 5°C in thecuvette holder as monitered *via* the instrument thermocouple. Initial AT+PIC (A) CD and (C) absorbance spectra were collected at 5°C. The temperature was subsuquently increased in increments of 5°C where the sample was remeasured until reaching a final temperatuer of 60°C. The AT+PIC sample was prepared in a 1 cm path length cuvette. The GC+PIC sample was prepared at room temperature and allowed to equilibrate to 2°C in the cuvette holder as monitered *via* the instrument thermocouple. Initial GC+PIC (B) CD and (D) absorbance spectra were collected at 2°C. The temperature was subsuquently increased in increments of 5°C where the sample was remeasured until reaching a final temperatuer of 22°C. The GC+PIC sample was prepared in a 0.3 cm path length cuvette.

The spectra demonstrate the susceptibility of the SA to changes in solution temperature (supported by correlation in the intensity change of the absorbance and CD peaks), while the J-bit peak is more stable. Though some red-shift of the CD and absorbance peaks was observed at greater temperatures (≥25 °C) the peak shape did not change much.

Supplemental AFM:

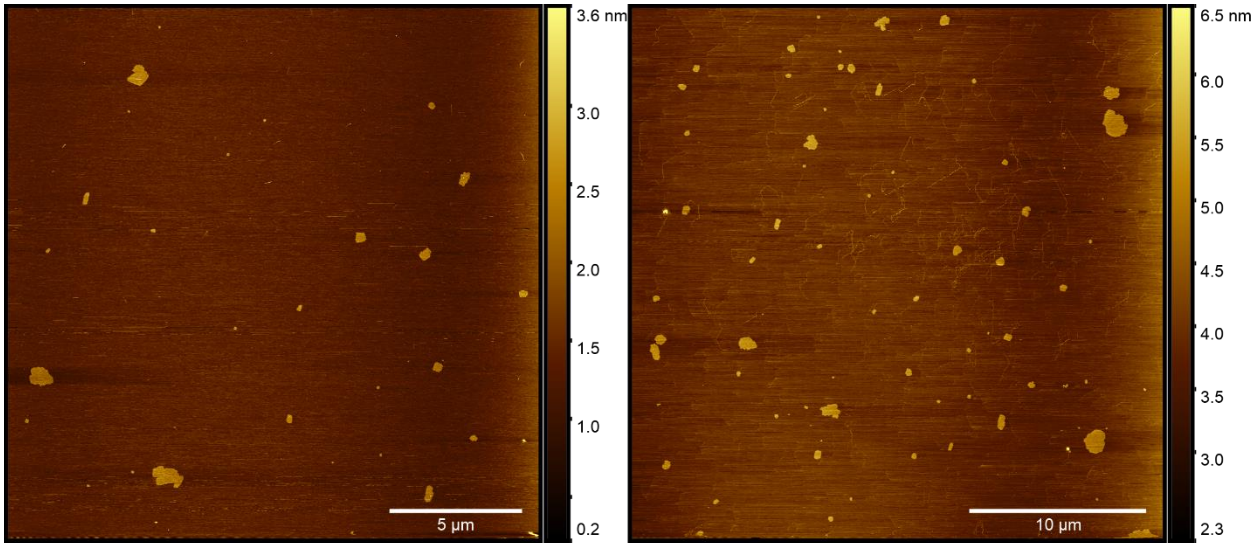

Figure S16: AFM images of PIC only control. AFM images captured of samples containing PIC only deposited onto mica substrate following the procedure outlined in the main text in *Materials and Methods*. **(Left)** Image of the PIC only control immediately after deposition on the mica substrate. (**Right**) Image of the PIC only control at the end of the measurement period (~45 minutes).

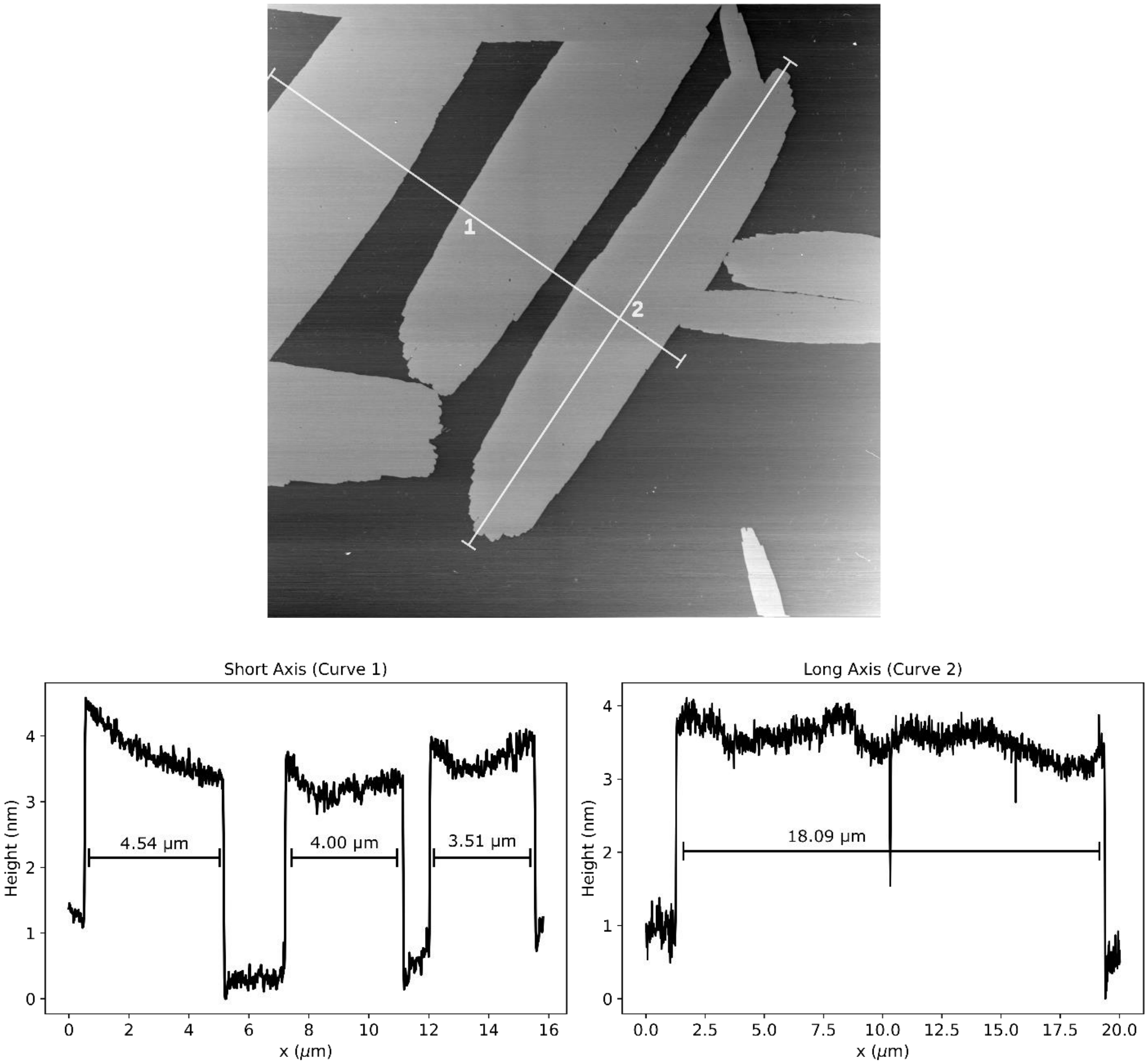

Figure S17: AFM image of SA formed with the AT DNA substrate. AFM image of a sample containing AT DX-tile and PIC deposited onto mica substrate following the procedure outlined in the main text in *Materials and Methods*. The numbered white lines correspond to the cross sectional profiles plotted below.

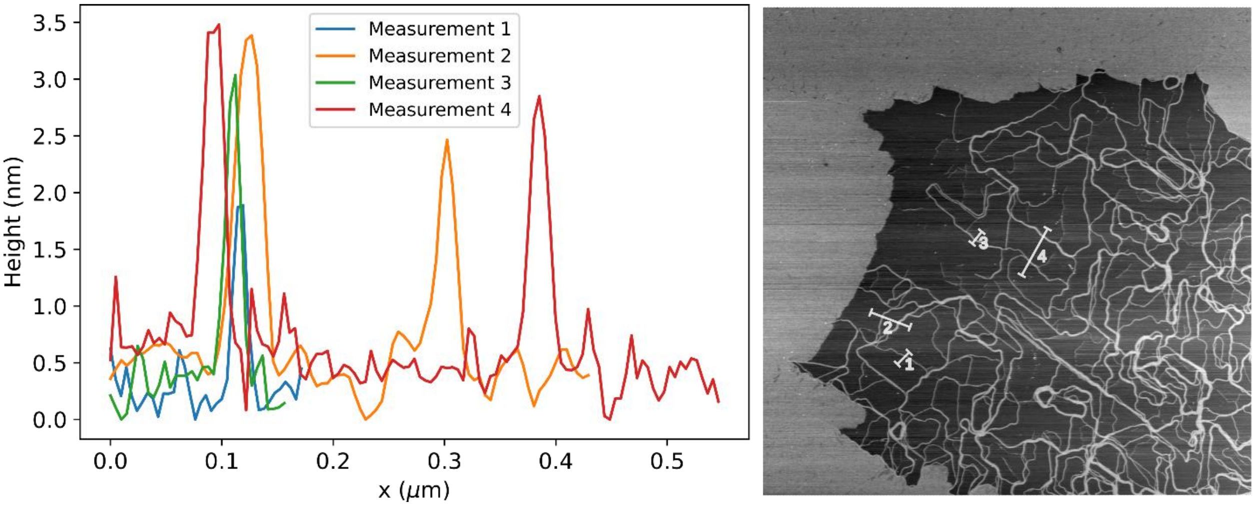

Figure S18: AFM image of SA formed with the dsDNA substrate. AFM image of a sample containing dsDNA and PIC deposited onto mica substrate following the procedure outlined in the main text in *Materials and Methods*. The numbered white lines correspond to the cross sectional profiles plotted left. The fibers are implied to be PIC as solution based PIC aggregates have been previously reported as fiber- or ribbon-like [4–6]. Furthermore, the height of the fibers appears to be ~2-5 nm which correlates closely with previously seen limits of PIC aggregation [7–9] as well as the larger structures seen in **Figure S17**.

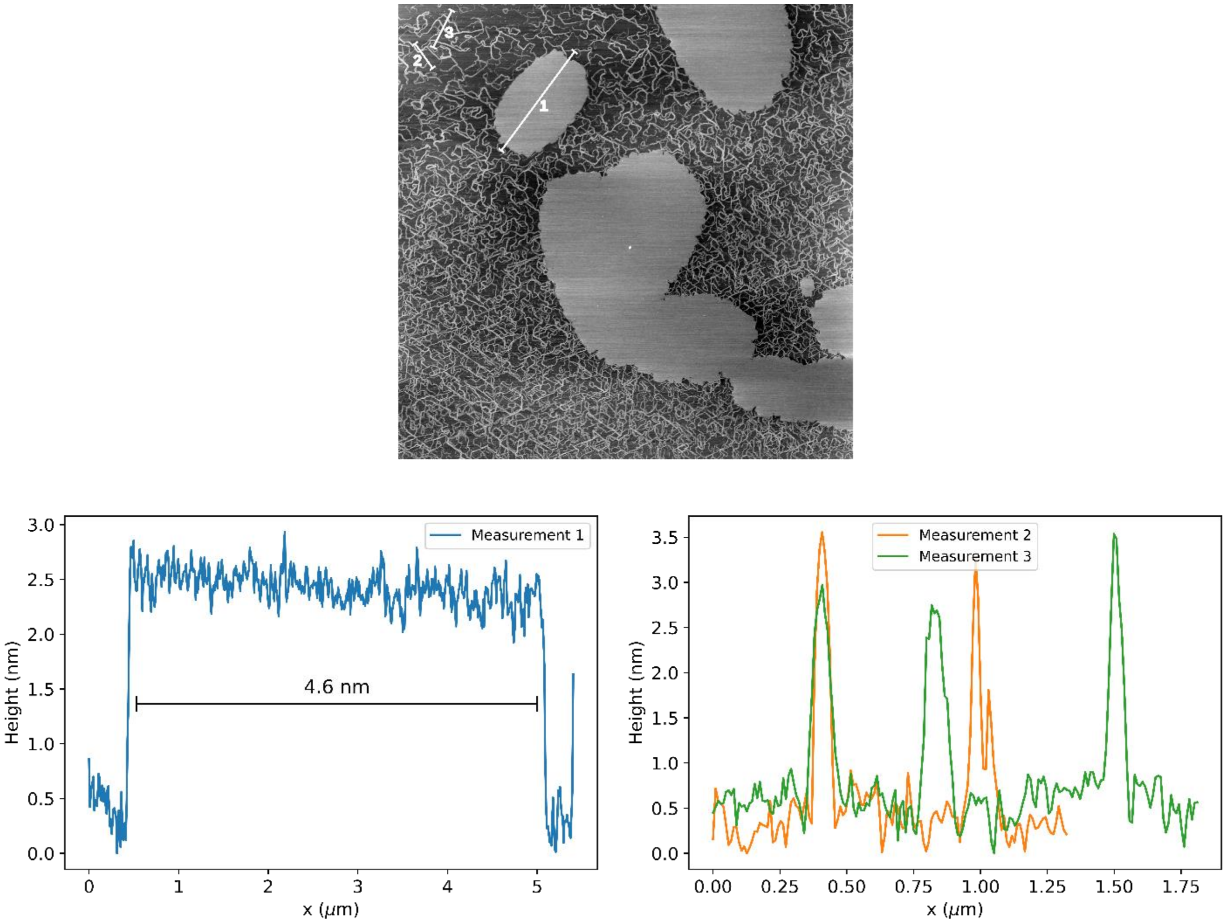

Figure S19: AFM image of SA formed with the ssDNA substrate. AFM image of a sample containing ssDNA and PIC deposited onto mica substrate following the procedure outlined in the main text in *Materials and Methods*. The numbered white lines correspond to the cross sectional profiles plotted below. The ssDNA sample appears to be the least stable as evidence by the least prolific aggregation. The leafy islands are more in line with the ones observed by Yoa, *et. al.* and Sugiyama, *et. al.* [7–10], approximately 2-5 nm thick and at least 4 nm in diameter (**bottom left)**. The PIC fibers are more pletiful and are consistent with those observed in **Figure S18.**

References:

1. Chiriboga M, Diaz SA, Mathur D, Hastman DA, Melinger JS, Veneziano R, et al. Understanding self-assembled pseudoisocyanine dye aggregates in DNA nanostructures and their exciton relay transfer capabilities. J Phys Chem B. 2021; 126:110–22.

2. Boulais É, Sawaya NP, Veneziano R, Andreoni A, Banal JL, Kondo T, et al. Programmed coherent coupling in a synthetic DNA-based excitonic circuit. Nat Mater. 2018; 17:159–66.

3. Banal JL, Kondo T, Veneziano R, Bathe M, Schlau-Cohen GS. Photophysics of J-aggregate-mediated energy transfer on DNA. J Phys Chem Lett. 2017; 8:5827–33.

4. Scheibe G, Kandler L, Ecker H. Polymerisation und polymere Adsorption als Ursache neuartiger Absorptionsbanden von organischen Farbstoffen. Naturwissenschaften. 1937; 25:75–75.

5. Daltrozzo E, Scheibe G, Gschwind K, Haimerl F. Structure of J-Aggregates of pseudoisocyanine. Photograph Sci Engin. 1974; (4):441–50.

6. Bricker WP, Banal JL, Stone MB, Bathe M. Molecular model of J-aggregated pseudoisocyanine fibers. J Chem Phys. 2018; 149:024905.

7. Yao H, Ikeda H, Kitamura N. Surface-induced J aggregation of pseudoisocyanine dye at a glass/solution interface studied by total-internal-reflection fluorescence spectroscopy. J Phys Chem B. 1998; 102:7691–4.

8. Ono SS, Yao H, Matsuoka O, Kawabata R, Kitamura N, Yamamoto S. Anisotropic growth of J aggregates of pseudoisocyanine dye at a mica/solution interface revealed by AFM and polarization absorption measurements. J Phys Chem B. 1999; 103:6909–12.

9. Yao H, Sugiyama S, Kawabata R, Ikeda H, Matsuoka O, Yamamoto S, et al. Spectroscopic and AFM studies on the structures of pseudoisocyanine J aggregates at a mica/water interface. J Phys Chem B. 1999; 103:4452–6.

10. Sugiyama S, Yao H, Matsuoka O, Kawabata R, Kitamura N, Yamamoto S. Three-dimensional structure of J aggregates of pseudoisocyanine chloride dyes at a mica/solution interface revealed by AFM. Chem Lett. 1999; 28:37–8.
